# Towards an enhanced indication of provisioning ecosystem services in agro-ecosystems

**DOI:** 10.1007/s10661-020-08816-y

**Published:** 2021-05-14

**Authors:** Claudia Bethwell, Benjamin Burkhard, Katrin Daedlow, Claudia Sattler, Moritz Reckling, Peter Zander

**Affiliations:** 1 Leibniz Centre for Agricultural Landscape Research (ZALF), Eberswalder Straße 84, 15374 Müncheberg, Germany; 2 Geography Department, Humboldt-Universität zu Berlin, Unter den Linden 6, 10099 Berlin, Germany; 3 Leibniz Universität Hannover, Institute of Physical Geography and Landscape Ecology, Schneiderberg 50, 30167 Hannover, Germany; 4 Division of Agriculture and Food Policy, Humboldt-Universität zu Berlin, Unter den Linden 6, 10099 Berlin, Germany

**Keywords:** Agricultural landscapes, Site-specific land use, Indicators, Management, Environmental effects

## Abstract

Provisioning ecosystem services play a vital role in sustaining human well-being. Agro-ecosystems contribute a significant share of these services, besides food and fodder and also fuel and fibre as well as regulating and cultural ecosystem services. Until now, the indication of provisioning ecosystem services of agro-ecosystems has been based almost only on yield numbers of agricultural products. Such an indication is problematic due to several reasons which include a disregard of the role of significant anthropogenic contributions to ecosystem service co-generation, external environmental effects and strong dependence on site conditions. We argue for an enhanced indication of provisioning ecosystem services that considers multiple aspects of their delivery. The conceptual base for such an indication has been made by prior publications which have been reviewed. Relevant points were taken up in this article and condensed into a conceptual model in order to develop a more holistic and expanded set of indictors, which was then exemplarily applied and tested in three case studies in Germany. The case studies represent different natural conditions, and the indicator set application showed that ecosystem services (ES) flow—in terms of output alone—does not characterise agro-ecosystems sufficiently. The proposed aspects of provisioning ecosystem services can give a fuller picture, for example, by input-output relationships, as it is possible by just using single indicators. Uncertainties as well as pros and cons of such an approach are elaborated. Finally, recommendations for an enhanced indication of provisioning ecosystem services in agro-ecosystems that can help to integrate agricultural principles with ideas of sustainability and site-specific land use are derived.

**Supplementary Information:**

The online version contains supplementary material available at 10.1007/s10661-020-08816-y.

## Introduction

### General background and objectives

The ecosystem services (ES) concept has gained great scientific importance especially during the last decade (Potschin et al. 2016), and the engagement of policy has also increased (Maes et al. 2012). For example, within its Biodiversity Strategy 2020^1^ (EC 2011), the European Union focuses very much on ES and recognises that biodiversity and functioning ecosystems are the base for ES supply. To create a knowledge base on ES supply, all member states were asked to map and assess the state of ecosystems and their services in their national territories until 2020 based on the Strategy’s Target 2 Action 5 (Maes et al. 2016). In 2016, the USA released a memorandum^2^ that directs agencies to incorporate ES into federal planning and decision-making. ES also find consideration in global sustainability policies such as the UN’s Sustainable Development Goals (SDGs; e.g. goal 15^3^) (Geijzendorffer et al. 2017).

ES are broadly defined as the benefits that nature provides to humans which are essential to their existence and well-being (Costanza et al. 1997; MEA 2005,4). ES include provisioning ES such as food, materials or energy and regulating and maintenance ES like climate, water and erosion regulation along with cultural ES such as recreational services (TEEB 2010,5; CICES^6^, see Haines-Young and Potschin 2013). A particular focus has been given to the generation of provisioning ES mostly in managed, to a lesser degree also in unmanaged, ecosystems (Plieninger et al. 2016), because these ES are at the core of direct human interest and activity, i.e. ensuring nutrition and material supply. There are different approaches to categorize provisioning ES (cf. MEA 2005; TEEB 2010; CICES, see Haines-Young and Potschin 2013). At the centre of most classification schemes is the biomass production from cultivated plants and animals, such as food and fodder biomass and raw materials. In addition, these ES provide genetic resources, medical and ornamental resources for humans and freshwater (TEEB 2010).

A fact that sets provisioning services apart from the other ES is that provisioning services often take the form of ecosystem ‘goods’, which actually can be more or less directly consumed or traded in markets, while for most of the other ES, such markets do not exist or function only poorly (Boyd and Banzhaf 2007). Moreover, in order to consider ecosystems that are managed based on anthropogenic system inputs, a newer definition of ES as ‘the contributions of ecosystem structure and function—in combination with other inputs—to human well-being (Burkhard et al. 2012a) has been proposed.

The MEA (2005, p. V) recognised that *people are integral parts of ecosystems*, and also the European Landscape Convention^7^ sees landscape as ‘an area, as perceived by people, whose character is the result of the action and interaction of natural and/or human factors’ (European Council 2000). This particularly applies to intensively used landscapes, including agro-ecosystems^8^. Agro-ecosystems and their provisioning ES strongly rely on the modification of natural ecosystems, with input-dependent energy, matter and information flows. Consequently, it does not seem realistic to consider only purely nature-derived goods as ES (Pérez-Soba et al. 2012). Therefore, we strongly support the idea to include co-generated outcomes (commodity products) of managed agro-ecosystems as ES and to indicate them jointly with positive and negative externalities of their supply.

Agro-ecosystems are managed ecosystems (Zhang et al. 2007; Power 2010). They are highly subjected to anthropogenic system inputs (‘agro-ecosystem services’, Burkhard et al. 2014), can affect multiple other ES (Zhang et al. 2007) and lead to positive or negative environmental impacts (Petz and van Oudenhoven 2012). Thus, both natural and human-derived capitals are needed for the co-generation of agro-ecosystem services (Jones et al. 2016). The supply of provisioning services, like the production of food market products, forage, fuel, pharmaceuticals or energy crops, belongs to the main objectives of agro-ecosystems (Power 2010; Kandziora et al. 2013a). But agro-ecosystems also provide regulation and maintenance ES such as climate and water regulation (e.g. Balmford et al. 2011) and cultural ES such as landscape aesthetics or knowledge systems (Huang et al. 2015). However, agro-ecosystems can also be the source of ecosystem dis-services (e.g. soil erosion (Steinhoff-Knopp and Burkhard 2018) and nitrate leaching (cf. Fridman and Kissinger 2018; Zhang et al. 2007)).

Also, as agricultural production strongly depends on other ES, it has been repeatedly mentioned as a user of (especially regulating) ES (Power 2016, 2010; Zhang et al. 2007). Pollination, pest and disease control, regulation of erosion, water, local climate and nutrients are the most common examples (Plieninger et al. 2016; Guerra and Pinto-Correia 2016; Guerra et al. 2016; Dungait et al. 2012). Societal goals are aimed at enhancing regulating ES and human well-being-supporting services, while strengthening the competitiveness of the agricultural sector. At landscape levels, this requires an enhancement of landscape multi-functionality (Rossing et al. 2009; Renting et al. 2009; Huang et al. 2015). Appropriate agricultural management strategies and decisions could enhance positive effects of agro-ecosystem services (e.g. Stein-Bachinger et al. 2015; Duru et al. 2015) and reduce negative environmental impacts (e.g. Zhang et al. 2007; Garbach et al. 2017; Kanter et al. 2018).

Looking at these complex interactions, emerging ES synergies and trade-offs, an indication of agro-ecosystem services should be able to answer questions like: ‘How can provisioning ES of agriculture be indicated?’, ‘How can the different forms of system inputs be distinguished and measured effectively to assess the above mentioned anthropogenic inputs and their resulting actual ES flows, and the positive and negative effects of their supply?’ and ‘How can this enhanced indication be applied on a regional scale?’.

An enhanced integrative indication of provisioning ES supply in agro- (and other managed) ecosystems improves the prevailing estimation of their (1) ES potentials, (2) anthropogenic inputs, (3) actual ES flows, (4) environmental externalities and (5) ES demands and preferences under consideration of the (6) mapping of provisioning ES. However, up to now, the third aspect has almost exclusively been widely discussed and actually used to measure provisioning ES (cf. Maes et al. 2016). Some promising conceptual attempts to overcome this research gap have been recently published. Jones et al. (2016) distinguished between stocks and flows of natural and human-derived capital, but remained on a rather conceptual level. Qualitative interview data have been used by Fischer and Eastwood (2016) to analyse ES co-production (and disservices) as human-nature interactions. Human inputs such as use of fertilisers, energy, irrigation, tillage or management knowledge have been considered relevant for agricultural ES supply by Albert et al. (2015) and Burkhard et al. (2014). Schröter et al. (2014) included the densities of cabins and of hiking paths, both can be considered anthropogenic inputs, to account for recreational cultural ES. Related approaches such as HANPP (human appropriation of net primary production, Haberl et al. 2012) calculate the human impact on ecosystem functionality and related ES supply and have been applied frequently. However, further efforts are required to close the research gap related to the inappropriate indication of provisioning ES.

With this study, we aim to fill this gap and explore and discuss additional indicators that cover the broad range of aspects listed above. Therefore, the overall objective is twofold: (i) to develop an enhanced indicator set for provisioning ES with an integration of the aspects: ES potentials, anthropogenic inputs, actual ES flows, environmental externalities, ES demands and preferences, spatial modelling and mapping of provisioning ES and (ii) to apply and test our enhanced indicator set in three different case study regions. We proceed as follows:

1. Elaborate the needs for new provisioning ES indicators in European and international policy and decision-making contexts.
2. Review and discuss existing indicators that relate to the different aspects with respect to their current applications, their merits and drawbacks.
3. Based on this discussion, develop and suggest an enhanced and more holistic/integrative indicator set which covers multiple aspects of provisioning ES.
4. Test this indicator set in three concrete case studies in Germany by applying a bio-economic farm model approach.
5. Discuss the new indicator approach and its case study application results, and look at uncertainties and pros and cons of its practical application.
6. Derive final recommendations for using such an approach in a wider European/international context in science, policy and practice, and indicate how far this approach contributes to understanding interrelations between agriculture, ecosystems and landscapes.

Hence, we will address the following four research questions:

- RQ1: Do we need a new rationale to describe provisioning ES supply realised as the combined outcome of the natural ecosystem potential and anthropogenic inputs under management?
- RQ2: Does a more holistic/integrative set of indicators, which includes all discussed aspects of provisioning ES, allow for a better indication?
- RQ3: Is this indicator set feasible for practical application at case study level (e.g. data needs and availability, measurability, comparability, revealing interdependencies, allowing valuation of environmental externalities, expendability by additional indicators)?
- RQ4: What are the implications for a wider application of the indicator set in a European/international context (e.g. transferability of data availability and of measurability)?

**Fig. 1:**
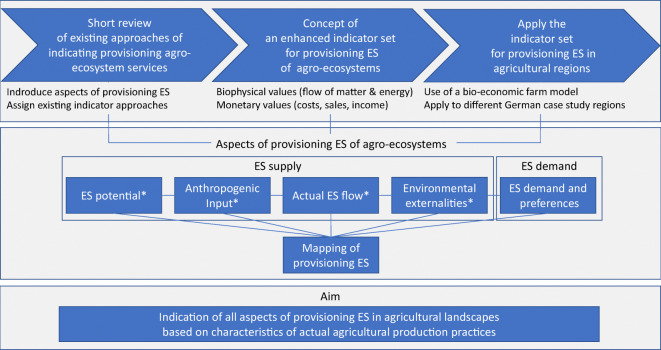
Overview of the main parts of the manuscript, the considered aspects of provisioning ES and the aim of the approach (*aspect is focused in the case study application)

Our key assumption is that outputs from agro-ecosystems are not ‘pure’ ES per se. Instead, they are highly influenced by anthropogenic system inputs, depend on other ES, result in environmental impacts and are bound to demands and preferences of markets and society. An integrative set of indictors as suggested in this study increases the quality of information for policy and planning of land use and agro-ecosystem management. For example, integrative indicators can help to enhance site-specific management of complex agricultural landscapes and related governance mechanisms. An overview of the main parts of the manuscript, the considered aspects of provisioning ES and the aim of the approach is shown in Fig. 1.

### European and international policy and decision-making contexts

ES have found a prominent place in current policy and decision-making, certainly at European Union (EU) and global levels. In the following, three examples are given in which an application of an enhanced indicator set of provisioning ES could be beneficial.

1. EU Biodiversity Strategy 2020

The EU Biodiversity Strategy 2020 is aimed at achieving more sustainable agriculture and forestry in the EU (target 3) and, at the same time, to halt the loss of global biodiversity and ES by 2020^9^. This includes the maintenance and restoration of ecosystems (target 2) and the improvement of knowledge of ecosystems and ES by Mapping and Assessing the state of Ecosystems and their Services (MAES^10^) within EU member states (action 5). Furthermore, the economic value of ES should be assessed and integrated into accounting and reporting systems at EU and national level by 2020 (EC 2011).

For provisioning ES from agriculture, Maes et al. (2014) suggested to use indicators such as yields of food, feed, fibre or energy crops (in t/ha); crop area (in ha); or produced amounts of biofuel, biodiesel or bioethanol (in kToe). They were aware that the proposed indicators did not consider that ES from agro-ecosystems are co-produced based on ecosystem and human management inputs. They furthermore asked for an agreed approach to discount human inputs (such as labour, machinery, irrigation, fertilisation, pest control) and to identify the contributions of ecosystems to production (Maes et al. 2014). Thus, the enhanced indication of provisioning ES from agro-ecosystems including environmental externalities as suggested in our study can help to bridge this gap and to go beyond commonly used agricultural production numbers as proxies. It remains to be tested how far data availability and quality hamper the enhanced indicator set’s implementation on EU and national levels.

2. European Agricultural Policy (CAP)

All current components of the Common European Agricultural Policy (CAP) contribute to compensate farmers for the costs of providing ES through agricultural activities (Baur and Schläpfer 2018). Recommendations for using an enhanced indicator set in the context of the CAP are given against the backdrop of scientific suggestions to improve the ecological effectiveness of environmental components (cross compliance, single and group contracted agri-environmental and climate measures (AECM), greening measures, e.g. ecological focus areas) of previous and current CAP periods (e.g. Batáry et al. 2015). On the other hand, such an indicator set could help to improve the implementation of the recently proposed programme for the next CAP period 2021–2027 (EU 2018; EC 2019,11; Jongeneel 2018). An enhanced indicator set can contribute science-based information and data to the current discussion of the CAP improvements, which can be summarised into the following five suggestions.

The first suggestion concerns the *integrated consideration of socio-economic and ecological aspects* as agro-ecosystem services. Agro-ecosystems link natural and human systems and the goods and services they generate (FAO 2014). The different aspects of our enhanced indicator set (see below) reflect this view. The integrated view on socio-economic and ecological aspects of agro-ecosystems (cf. Mouysset 2017) is essential for development, adaptation and valuation of AECM. An integrated view can ensure that these measures are economically feasible for farmers, as a main driver for farm decision-making processes (Wolters et al. 2014) and that sustainable management practices can be adapted to site conditions, mitigate environmental impacts and fulfil rising societal demands for environmentally friendly produced agricultural goods.

The second suggestion argues for a *better targeting of measures*. Environmental objectives (cf. Meyer et al. 2015) and spatial targeting (cf. Reed et al. 2014; Ekroos et al. 2014) mean to set priorities for ES and biodiversity targets in particular areas for applying corresponding measures. Environmental objectives are targeted in the case of expected promising results and effective application of expenditures to maintain or restore these results. The spatial targeting can consider the variability of biophysical conditions, management costs, potentials to deliver expected results from environmental measures and the appropriate scale and boundary of the targeted environmental objectives (Reed et al. 2014). An enhanced indicator set can be used to identify objectives and appropriate areas, due to the capacity of indicators for inter- and intra-regional comparisons.

The third suggestion, *the integration of all measures at the landscape level*, is seen by many authors as a crucial step to reach ES and biodiversity objectives (cf. Leventon et al. 2017; Lefebvre et al. 2014; Prager et al. 2012). This requires the collaboration between different stakeholders (e.g. Prager and Freese 2009; Prager 2015) and the application of systemic approaches (e.g. Lescourret et al. 2015). This has already been implemented in several European countries (e.g. Westerink et al. 2017a; de Krom 2017; Franks and Emery 2013). Local knowledge on farm, landscape or regional levels (cf. Zasada et al. 2017) can help to achieve superior ES and biodiversity objectives (e.g. McKenzie et al. 2013) or to contribute to a green infrastructure (e.g. Maes et al. 2015; Schmidt and Hauck 2018). Interactions between on- and off-field areas are complex and depend in detail on landscape structures and the targeted species (e.g. Concepcion et al. 2012), supporting the idea of integration. Significant positive effects on regulating (e.g. Westerink et al. 2017b) or cultural (e.g. van Berkel and Verburg 2014) ES can be achieved by collaboration in a landscape with a clear common vision and a local facilitator (e.g. Prager 2015). Especially biodiversity and climate change targets are suitable to derive multifunctional effects by coordination (Galler et al. 2015), but there are also limits in providing multiple ES (Maskell et al. 2013). That means, regional aims should be carefully defined by experts and local stakeholders, e.g. by hybrid governance approaches (e.g. Velten et al. 2018).

The fourth point bundles suggestions which require *continuous information sampling for evidence-based decision-making* on different levels and which *stimulate ongoing learning processes* amongst stakeholders and decision-makers. These suggestions include (i) introducing more result-based measures (cf. Plieninger et al. 2012; Burton and Schwarz 2013; Herzon et al. 2018; Reed et al. 2014; Russi et al. 2016), (ii) accompanying measures by monitoring (e.g. Prager 2015), (iii) allowing flexible application of measures and introducing adaptive management (cf. Meyer et al. 2015; Hodbod et al. 2016), (iv) fostering farm advisory and knowledge-exchange (e.g. Meyer et al. 2015; Schomers et al. 2015) and (v) developing and applying knowledge and innovation systems (e.g. Bommarco et al. 2018). Based on these points, an appropriate information stream can enable farmers to apply measures in a flexible way and to adapt to changing frame conditions within viable agricultural production systems. The enhanced indicator set provides a framework that accompanies such information-based processes.

The fifth suggestion is to find *tailor-made solutions* for the EU member states at the regional and national levels, because agri-structural characteristics and natural conditions differ between the member states and cause various conditions for an implementation of an overall programme in the countries (cf. Öhlund et al. 2015) and regions (cf. Kirchner et al. 2016). The application of an enhanced indicator set can help to identify specific tailor-made solutions at different organisational levels and thereby support the aim to sustain the diversity of European agricultural landscapes at various scales (c.f. Lefebvre et al. 2014).

3. Ecosystem/natural capital accounting

Ecosystem/natural capital accounting is aimed at integrated assessments of human-environmental interrelations by measuring ecosystems, their condition and ES flows from ecosystems into economic and other human activities (SEEA EEA^12^; Science for Environment Policy 2017,13). The System of Environmental Economic Accounts (SEEA) is connected to the Systems of National Accounts (SNA) and part of the statistical systems in many countries of the world. Steps of ecosystem accounting include assessing ecosystem extent (e.g. agricultural land area), ecosystem condition, ES supply and use and monetary ES assessments. SEEA also recommends to consider disservices that emerge from agricultural land use. The overall aim is to understand the dependence of economic activities on ecosystems and their condition. Besides the SEEA framework, detailed SEEA guidelines for practical applications were developed on EU level (Science for Environment Policy 2017) and globally (United Nations 2014). SEEA and SNA recognise that cultivated systems are managed systems with high human inputs. To properly define and ensure consistency with the (agricultural) production boundary, (natural) ecosystem contributions must be distinguished from (cultivated) anthropogenic production inputs. The existing SEEA guidelines are currently under revision, and the suggested enhanced indication of provisioning ES in agro-ecosystems can help to develop applicable indicators.

### Existing approaches of indicating provisioning agro-ecosystem services

Agro-ecosystem services, their provision and potential indicators for their quantification have been studied intensively. However, to understand the relations between ecosystems and agriculture, we should not only look into the production process with the potential of agro-ecosystems to deliver provisioning ES (the related anthropogenic inputs and resulting actual provision, e.g. yields) but also analyse the positive and negative externalities of agricultural land use on ecosystems. The spatio-temporal phenomena of these aspects are important, and they can be assessed by spatial modelling and mapping of provisioning ES. As the valuation of these impacts depends on the perception of consumers, regional stakeholders and society at large, the relation between agricultural production and the ES preferences and demands also plays a role. Therefore, we distinguish between six different aspects for the indication of provisioning ES and suggest specific approaches for their assessment:

#### ES potential of agro-ecosystems

The natural potential to deliver provisioning ES varies substantially between different agro-ecosystems. Site conditions, like radiation and temperature, the site-specific long-term soil quality in terms of productivity (depending on soil types and characteristics) and crop features are all factors that define the potential of an agro-ecosystem to generate crop biomass (according to Duru et al. 2015, completed by site-specific soil quality). Limiting abiotic factors (e.g. water, nutrients) and reducing biotic factors (e.g. weeds, pests, diseases) can be compensated by anthropogenic inputs. However, the assessment of the input shares of the ecosystem-based ES potential and anthropogenic inputs is challenging. Therefore, assessments are often aimed at the overall potential for agricultural provisioning ES, by assigning site-specific, mostly natural, relative differences in soil and climate characteristics, for example, by soil quality ratings (cf. Bünemann et al. 2018). One example is the soil quality rating index (Müller et al. 2007: M-SQR-Index), used also as part of national natural capital accountancies (Albert et al. 2016). Further indicators are used that report on the sensitivity and actual condition of agricultural ecosystems which can increase or reduce soil productivity, e.g. in respect to soil biota (Barrios 2007; Griffiths et al. 2018), pest control (Chaplin-Kramer et al. 2013) or levels of soil erosion (e.g. Meyerson et al. 2005).

#### Anthropogenic inputs

The provisioning services of agro-ecosystems are the result of anthropogenic inputs in combination with natural ecosystem conditions. The actual ES flow can be assigned to various shares of natural and anthropogenic inputs (Duru et al. 2015). Anthropogenic inputs from an ecosystem perspective (Kandziora et al. 2013b) result in changes of the balances of energy, water and matter and also in structural variables. They are embedded in a specific management system, dependent on the aims of the farming activities and the site conditions, different cultivated crops, crop rotations and locally adapted management practices (e.g. tillage/non-tillage operations, fertilisation practices, pest and disease management). Thus, the whole management system can be seen as an external, anthropogenic input that heavily influences the characteristics of an agro-ecosystem (Pérez-Soba et al. 2012). Furthermore, legacy effects can occur, when previous input management strategies still have an effect over time (cf. Rutgers et al. 2012). Often, more unspecific terms like low vs. high intensity (e.g. Tamburini et al. 2016), intensity gradients (e.g. Syswerda and Robertson 2014) or ecological vs. conventional farming (e.g. Williams and Hedlund 2013; Batáry et al. 2012) are used to indicate anthropogenic inputs. However, a more detailed focus on the effects of specific management practices on the provision of ES would help to develop sustainable management strategies, as concluded by Williams and Hedlund (2013). Indicators are available which give more detailed insights into management practices, for example, for pesticide application, as used by Sattler et al. (2007: standardised treatment index). The development of indicator sets, which allow (i) to assess the consumption of energy, water and other resources (nitrogen, pesticides, machinery) per produced unit, i.e. to assess them by intensity indicators (cf. Ruiz-Martinez et al. 2015), (ii) to assess balances (e.g. GHG-balances) and (iii) to compare different production systems regarding their inputs and related ES, i.e. efficiency indicators (e.g. energy and water use efficiency), should accompany management strategies for innovative, resource-efficient solutions, which are embedded in the agricultural system (cf. Wolters et al. 2014).

#### Actual ES flow from agro-ecosystems

In order to indicate realised ES flows**,** a strategy of three steps was suggested by Meyerson et al. (2005): assess (i) the extent of an ecosystem, (ii) the condition of an ecosystem and (iii) quantities of some flows of ecosystem-oriented goods. Following this logic, land use/cover data has been used as a first proxy or a capacity estimation for actual ES flows (e.g. Burkhard et al. 2009, 2012b, 2014). However, this proxy exposes uncertainties (Hou et al. 2013) and should be completed (Van der Biest et al. 2015) by more detailed assessments (cf. Meyerson et al. 2005). Often, crop and grassland cultivated areas or crop yields (tons/energy per year and unit land) and livestock data (numbers or livestock units per unit land, tons/energy per year and region) (e.g. Fridman and Kissinger 2018; Kandziora et al. 2013a; Balbi et al. 2015) are primarily used for indication (Maes et al. 2016). Only a few studies use aggregated indicators like grain equivalent units (Koschke et al. 2013), which allow to compare agricultural production between regions. However, it is arguable whether such an indication, solely through used area and achieved yields, is adequate without taking anthropogenic inputs and external impacts into account, because it may not comply with the basic idea of the ES concept, which is to safeguard natural capital while maintaining sustainable flows of ES from nature to society (Burkhard et al. 2012a). Furthermore, the net primary production (cf. Haberl et al. 2012: NPP; e.g. Kandziora et al. 2013a) was used. A few studies include farm economic indicators, like sales and farm income (e.g. Crossman and Bryan 2009; Koschke et al. 2013; Kirchner et al. 2015; Firbank et al. 2018), but the factor costs, although necessarily assessed, because the farm income is based on a difference between sales and costs, are not explicitly described in most studies. In case the factor costs were made explicit, they belonged to the aspect anthropogenic input. An energy balance approach for the assessment of the ES flow of agro-ecosystems was suggested by Pérez-Soba et al. (2012). Indicators representing quality aspects of provisioning ES were applied for forage production (e.g. Van Vooren et al. 2018) and orchards (e.g. Demestihas et al. 2017). The quality aspects of crop production (cf. Wang et al. 2008), although important for the establishment of product prices, have not yet been represented by provisioning ES indicators. Other indicators include the actual amounts of marketed products for consumption (e.g. harvested biomass actually sold, cf. Geijzendorffer et al. 2017). Sometimes, these figures were corrected by the amounts of products that are spoiled and disposed of before consumption (e.g. Rasmussen et al. 2016).

#### Environmental externalities of provisioning ES

An ‘informed management’ emphasizes the mitigation of negative environmental impacts and the enhancement of regulating and cultural ES (Pérez-Soba et al. 2012). To assess the environmental impacts of provisioning ES, either positive (win-win/synergy; e.g. Bareille and Letort 2018; Daryanto et al. 2018; Everwand et al. 2017) or negative (trade-off; e.g. Gissi et al. 2018) externalities of production are considered (e.g. Bennett et al. 2009; Howe et al. 2014). For an assessment of environmental externalities, the identification and operationalization of indicators are two important steps (Kanter et al. 2018). Indicators cover a wide variety of possible externalities on different spatial scales (e.g. Williams and Hedlund 2013; Balbi et al. 2015; Schulte et al. 2014). For regulating ES, mainly information of soil-related ES is available (e.g. soil erosion control, nitrogen fixation), followed by indicators for pollination (e.g. pollinator abundance, pollination potential), whereby for cultural ES (e.g. rural tourism), only few indicators are available (Maes et al. 2016). Often, the externalities are assigned to broad categories of agricultural production, but their assessment should focus on specific management practices (Williams and Hedlund 2013), e.g. Garbach et al. (2017) and Techen and Helming (2017). As the scope of possible externalities is large, analysis must focus on a number of well-defined externalities that depend on societal perception (Rodríguez et al. 2006; Kroeger 2013). Generally, most assessments are based on agricultural intensity indicators (cf. Firbank et al. 2018), whereby site conditions, management practice, ES flow and site-specific sensitivity are all important determinants for environmental impacts (e.g. Albert et al. 2016; Sattler et al. 2010; Tsonkova et al. 2015).

For an assessment of externalities of provisioning ES for biodiversity aspects and regulating and cultural services within agricultural landscapes, the landscape structure in general (e.g. Kleijn et al. 2011), specific landscape elements (e.g. Firbank et al. 2018), interrelations between them and management practices (e.g. Tamburini et al. 2016) and their relations to site-specific sensitivities should be considered. For biodiversity aspects, these facets should be related to the habitat requirements of taxonomical and functional species groups (e.g. Liere et al. 2017; Birkhofer et al. 2018). Thus, the development of indicators is challenging and assessments often use proxies (e.g. Andersen et al. 2013) and/or scorings (e.g. Firbank et al. 2018; Tzilivakis et al. 2016; Overmars et al. 2014). Regulating ES that are used by farmers (e.g. natural pest control, pollination, soil erosion control) require accurate specific indicators (e.g. Rusch et al. 2012; Steinhoff-Knopp and Burkhard 2018) and are ideally related to landscape patterns (e.g. Duarte et al. 2018). Broader assessments of environmental quality are part of farm performance evaluations (e.g. Firbank et al. 2018) or describe developments of agricultural landscapes (e.g. Björklund et al. 1999).

#### ES demand and preferences

That ES supply is driven by demand holds especially true for the actual ES flow of provisioning ES from agro-ecosystems, which are mainly marketed products. Thus, this demand is related to consumer interests. Positive externalities of agriculture are usually driven by regional stakeholder or societal interests, e.g. provisioning of habitats for or of cultural ES of agricultural landscapes. Also, there is a demand to align the agricultural production in order to mitigate their negative externalities. Consumer, regional stakeholder and societal demand and preference assessments that regard provisioning as well as regulating and cultural ES from agro-ecosystems, their functions and structures help stakeholders (amongst them farmers) and politicians to decide on suitable agricultural management strategies to support the ES in high demand. Such integrated management strategies consider that agricultural products and non-marketed ES are jointly produced (Kragt and Robertson 2014; Tsonkova et al. 2015; Huang et al. 2015; Klapwijk et al. 2014).

Demand and preferences for consumer interests can be indicated through analyses of consumption patterns (i.e. consumption rates related to population density, cf. Villamagna et al. 2013). Additionally, the type or quality of marketed products is often used for indication at the individual level (e.g. share of organic and regional products, cf. Rödiger and Hamm 2015; Feldmann and Hamm 2015; Hempel and Hamm 2016). Willingness-to-pay (WTP) or willingness-to-accept (WTA) studies, employing stated or revealed preference analyses, can also be used for assessing regional stakeholder interests. These studies have to deal with the difficulty that people do not always recognise the capacity of ecosystems to provide ES (Martín-López et al. 2012). Demand and preferences are also influenced by availability of appropriate substitutes (cf. Rasmussen et al. 2016). Scarcity is another issue which is relevant in this context, as scarce resources are typically in higher demand (cf. Meyerson et al. 2005). Scarcity is often reflected in market prices driven up by high demand (e.g. Geijzendorffer et al. 2017).

Farmers and other regional stakeholders can benefit in manifold ways from specific locally provided ES, and these provided ES can, for example, influence the farmers’ perception of ES (Smith and Sullivan 2014; Teixeira et al. 2018). Generally, an individual demand of regional stakeholders can be quantified by methods like food diaries, group interviews, participant observations or surveys (Rasmussen et al. 2016) in specific studies or panels. The demand to mitigate negative externalities can be assessed by the amount of needed regulation to meet a desired environmental quality (cf. Villamagna et al. 2013). From an ecosystem perspective, the ecological work which is needed to achieve the socially defined environmental quality under a given ecological pressure determines the needed regulating service flow and depends on the regulating capacity of the ecosystem (Villamagna et al. 2013).

Apart from expressed demand at the consumer and regional stakeholder level, demand can also be expressed at the societal level through existing agri-environmental policies (cf. Schulte et al. 2014) or policies aiming at avoiding environmental risks (Wolff et al. 2015, 2017). These policies define indicators specific to their aimed and regularly reported environmental goods (e.g. Water Directive 2000 (EC 2000)^14^, fauna flora habitat directive (EC 1992)^15^).

Relations of ES demands to ES supply could be expressed as an ES footprint, defined as the area needed to generate particular ecosystem goods and services demanded by humans in a certain area at a certain time (Burkhard et al. 2012b). There are also studies which show the spatial variation of ES demand through mapping approaches (cf. Wolff et al. 2017). ES demand is, compared to ES supply, still insufficiently researched (cf. Geijzendorffer et al. 2017).

#### Spatial modelling and mapping of provisioning ES

As a cross-cutting issue, all indicators discussed above (aspects 1–5) show spatial and temporal variations. This can be best visualised by spatial modelling and mapping of provisioning ES. To show the spatial variation, single thematic ES maps can be used. To highlight temporal variations, a whole series of maps displaying changes over time is more appropriate (e.g. to show seasonal or annual variability). In dependency on the ES in question, different resolutions might be adequate.

For spatial modelling and mapping of provisioning ES supply, different data sources and methods can be used (Burkhard and Maes 2017). This includes look-up tables, based, for instance, on aggregated statistics and spatial interpolation, which are often used to display actual ES flow mapping (e.g. maize equivalent yield). Furthermore, causal relationships and environmental regression approaches, which are often used for regulating and cultural ES, can be used to assess environmental externalities (e.g. sequestered carbon) (Schröter et al. 2015). In a few studies, spatial ES supply concentration was indicated by assessing hot or cold spots (e.g. Früh-Müller et al. 2016) or temporally high ES supply can be indicated as a hot moment (Burkhard et al. 2014).

In principle, spatial modelling and mapping of ES can be done for all indicators described for the different aspects discussed above in sections of aspects 1–5.

An example for a mapped *ES potential* can be found in Albert et al. (2016), who suggest to use the yield potential of arable soils (BGR 2013) based on Müller et al. (2007) for national accountancies. *Anthropogenic inputs* are mapped mainly by intensity indicators, for example, the management intensity of grasslands (Estel et al. 2018) or agricultural land (van der Zanden et al. 2016) at a European scale. Mapped crop rotations (Koschke et al. 2013) depict the time and organisational processes of farms (Grunewald et al. 2013) and are important for the extent of environmental externalities, e.g. soil erosion (Guerra and Pinto-Correia 2016), habitat quality (Glemnitz et al. 2015). *Actual ES flows* in terms of crop yield and yield uncertainties are mapped on a regional scale (e.g. Balbi et al. 2015). A mapping of *environmental externalities* of provisioning ES considers trade-offs to regulating (e.g. Balbi et al. 2015), habitat (e.g. Willemen et al. 2012) or cultural ES (e.g. van Zanten et al. 2016). On an agricultural landscape scale, trade-offs between provisioning ES and habitat or cultural ES can be analysed by using landscape metric indicators, i.e. fragmentation, diversity, habitat connectivity, habitat richness and landscape heterogeneity (e.g. Frank et al. 2012; Koschke et al. 2013; Duarte et al. 2018; Kay et al. 2018). Geostatistical indicators can be used to explore patterns of ES (Ungaro et al. 2014, 2017). *ES demand* is regarded only by a few studies that for instance indicate a mismatch between ES supply and demand by matrix approaches, considering also provisioning ES (e.g. Burkhard et al. 2012b, 2014). Mostly, ES maps refer to the spatial variability of ES with only a few approaches that consider the temporal variability of ES supply (cf. Burkhard et al. 2014).

#### Overall indicators

In addition to the above listed indicators, also combined approaches which cover several aspects have been suggested. For instance, the EBI (Ecosystem service Bundle Index) was introduced by Van der Biest et al. (2014) to combine biophysical (ES supply-oriented) and socio-economic (ES demand-oriented) aspects.

When using indicators of all aspects, the following must be kept in mind: to avoid misinterpretation of indicators, it is important to explicitly describe the metadata of the analysis, e.g. objective, system-boundaries; characteristics of used data (data preparation, data resolution, data assessment aim); methods; uncertainties (e.g. Grêt-Regamey et al. 2014; Schulp et al. 2014) and scale (Hein et al. 2006; Haines-Young et al. 2012). For example, system boundaries play a key role in several aspects of provisioning ES. An example for a spatial boundary is that the results of GHG-emission of a production process differ between a calculation which only takes into consideration the emissions which occur during management on a field and a calculation which additionally considers the emissions in the upstream chain (e.g. mineral fertiliser production). The temporal boundary is related to the fact that crops are affected not only by management strategies and climate during the growing period but also by long-term soil processes related to the production of previous crops (Angus et al. 2015; Preissel et al. 2015). Thus, an assessment at the crop rotation scale (i.e. the rotation is the temporal system boundary) allows to quantify crop yield (which belongs to actual ES flow) while considering also long-term processes (Reckling et al. 2016).

## Development of the enhanced indicator set for provisioning ES of agro-ecosystems

It is obvious that the generation of provisioning ES in human-modified agricultural land use systems is strongly dependent on natural and human-derived inputs (Jones et al. 2016; Power 2016). Respective indicator systems should therefore reflect these aspects by distinguishing between natural and anthropogenic contributions as well as by informing about environmental impacts. They should furthermore regard interests of consumers, regional stakeholders and the whole society, as well as describe spatio-temporal phenomena.

### Conceptual model

**Fig. 2:**
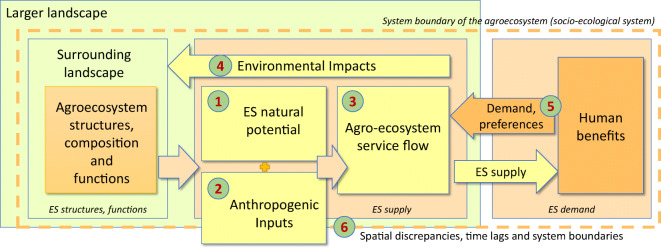
Conceptual model of ecosystem service co-production in agro-ecosystems (according to Burkhard et al. 2014)

The conceptual model in Fig. 2 gives an overview of ES flows from agro-ecosystems to society (after Burkhard et al. 2014; Bastian et al. 2013; and the ES ‘cascade’ model by Potschin and Haines-Young 2016). The role of anthropogenic inputs and ES potentials is illustrated in the left part of the ‘ES supply box’ in the centre. Thus, the ES potential is enhanced (as in the case of provisioning ES in agro-ecosystems) by anthropogenic inputs, activating a flow of usable ES that eventually benefit the human population and economic activities, which demand certain ES. The harnessing of ES and especially the optimisation of the supply of selected provisioning ES in agro-ecosystems based on anthropogenic inputs lead to trade-offs between ES, degradation of natural capital and environmental impacts (Rodríguez et al. 2006). The identification, quantification and assessment of trade-offs (and synergies) between ES and environmental impacts of human land use systems are one of the key strengths and a major application potential of the ES concept (Foley et al. 2005).

Suitable indicators are needed to quantify and to communicate the relevance and effects of the different components in the conceptual model. In this article, we will focus on (1) ES potentials, (2) anthropogenic inputs, (3) actual ES flows, (4) environmental externalities of provisioning ES, (5) ES demands and preferences and (6) spatial modelling and mapping of provisioning ES in agro-ecosystems by using the example of food provision. In most of the currently available provisioning ES indicator sets, the aforementioned components are summarised in one indicator, which normally is ‘yield in tons/area and time’ (see ‘Introduction’, part three, aspect three).

**Fig. 3:**
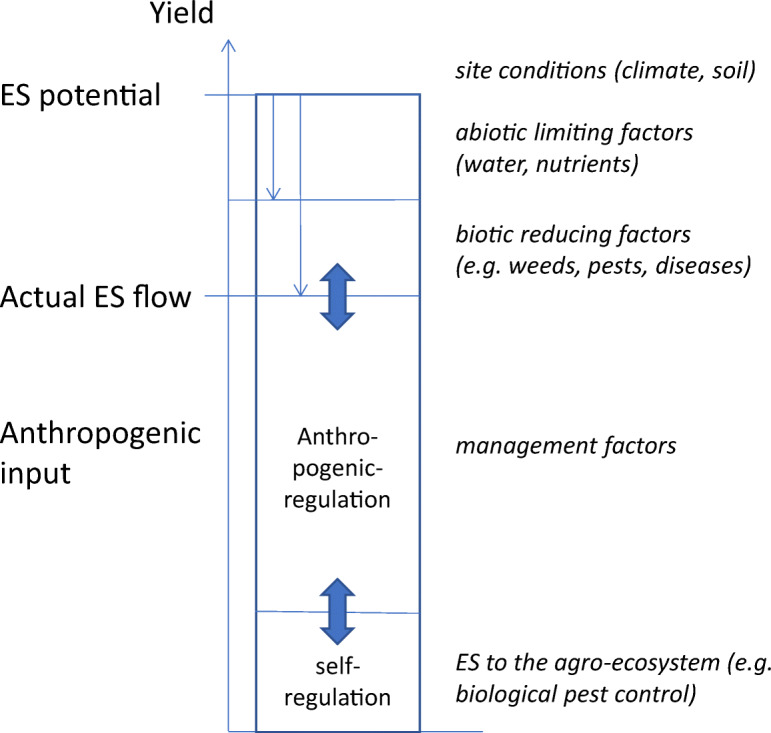
The distinction between the ES potential, the ES flow and the anthropogenic input (adapted from Duru et al. 2015)

The distinction between ES potential, anthropogenic inputs and actual ES flow, altogether the central part of our conceptual model, is shown in Figure 3 (according to Duru et al. 2015, see also ‘Introduction’ part three, aspects one to three). In the conceptual model, we refer to an overall ES potential, defined by the site conditions (such as climate, long-term soil quality) and crop features. In a long-term perspective, the ES potential can be enhanced, for instance based on technological developments. The actual ES flow is defined by the ES potential, which can be limited by abiotic (e.g. water, nutrients) and biotic (e.g. weeds, pests, diseases) factors and which depends on the choice of the crop genotype and management as anthropogenic inputs. Furthermore, the choice of crop and land management strategies are important factors which determine whether and to which degree the agro-ecosystem can be considered self- or anthropogenic-regulated and how large the environmental impacts are.

### Requirements to indicators of the enhanced indicator set

**Fig. 4:**
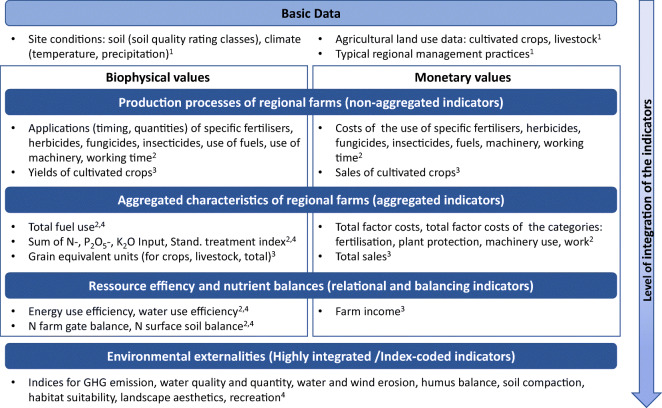
Data and levels of integration of the indicators for provisioning ES, example for cultivated crops for human nutrition: hierarchical scheme (^1^indicators of ES potential, ^2^indicators of anthropogenic input, ^3^indicators of ES flow, ^4^indicators of environmental impact)

The following requirements are needed for indicators of the introduced aspects of provisioning ES (see ‘Introduction’, part three). Different levels of indicator integration are necessary to fulfil the requirements of those indicators, which refer to the ES supply (aspect one to four) and demand (aspect five). The levels of integration of the indicators range from detailed biophysical and socio-economic data on the site condition and farming activities up to complex and highly integrated data on environmental externalities (Fig. 4). Most of those indicators can be expressed by biophysical, non-monetary values to represent the ecological side of ES generation and to quantify the flow of matter and energy (see left side, Fig. 4) and by monetary values to represent the economic side and to quantify costs, sales and income (right side, Fig. 4).

First, to achieve transferability of the indicators, the basic datasets should ensure that the indicators are based mainly on quantitative, harmonised, available and if possible, spatially explicit datasets which meet the requirements of the ‘high-quality data label’ of ES indicators according to Maes et al. (2016). In the following sections, such data are referred to as ‘data of high-quality requirements’. That means regional analyses of agricultural provisioning ES start with (i) datasets that describe site conditions to define the ES potential, based on widely accessible geodata, and (ii) the crop cultivation and livestock data which are, for example, available from agricultural census or from the Integrated Administration and Control System (IACS)^16^.

Second, a detailed regional assessment of the management practices can be achieved by quantitative, non-aggregated indicators which are used to describe the anthropogenic inputs and the actual ES flow. The indicators are based on regional cultivated crops, livestock and management practices and can be expressed as biophysical values to assess the input (e.g. fertiliser products, amount and times of fertilisation, pesticide products, amount and times of pesticide application, use of fuels, machinery, applied working time) and the actual ES flow (e.g. crop yields), or as monetary values to assess the input (e.g. costs of inputs) and the actual ES flow (e.g. crops sales). For the anthropogenic input, not only the mentioned direct inputs but also indirect inputs (e.g. educational level of farmers) should be considered.

Third, to achieve comparability between different regions, quantitative, aggregated indicators are needed. Aggregated indicators can assess the anthropogenic input and the actual ES flow (as output), also in the case of regional different crop patterns and animal husbandry. The input and output can be made comparable through aggregated budgeting of provisioning ES. That means, for example, in terms of biophysical values to calculate aggregated numbers of fertilisation, grain equivalent unit yields and in terms of monetary values to calculate total factor costs and total sales.

Fourth, to relate input and output of agricultural production, quantitative, relational indicators can be used. They relate output to input, i.e. actual ES flow to anthropogenic input or express balances, i.e. N balances. Indicators, for example, expressed in biophysical values, are energy and water use efficiency and, in monetary values, farm income. Indicators of this category can mainly be used to assess resource efficiency and show ways for reducing environmental impacts and simultaneously maintain or improve actual ES flows.

Fifth, highly integrated and index-coded indicators can be used to assess environmental impacts. Ideally, these indicators are based on applied management practices to ensure that environmental externalities of management practices can be compared and implemented and that environmentally friendly practices can be monitored by the indicators.

Sixth, indicators for ES demand and preferences can reflect consumer interests and local and regional stakeholder interests—in this case, they are often derived from stakeholder processes or have a normative character to fulfil societal demands.

### Enhanced indicator set

The comprehensive indicator set for assessing provisioning ES from agro-ecosystems captures the aspects that are described in the following. The respective indicators related to these aspects are presented in Table 1 and follow the requirements defined in the previous section. Major parts of the indicator set were utilised in the case study applications (Table 1 in bold).

**Table 1:** Indicators for provisioning ES, example for cultivated crops for human nutrition (indicators in bold were quantified in the case studies)

Supply side				Demand side
1. ES potential	2. Anthropogenic inputs (as biophysical and monetary values)	3. Actual ES flow (actual provision as biophysical and monetary values)	4. Environmental externalities of provisioning ES (positive, negative)	5. ES demands and preferences
6. Spatial modelling and mapping of provisioning ES				
Soil and climate conditions - Soil quality rating (index)^1a,b^ - Temperature^1b^ - Precipitation^1b^	Direct inputs: Non-aggregated biophysical and monetary values - Seeds^2a^ - Fertiliser^2a^ - Pesticides^2a^ - Energy (fuel consumption)^2a^ - Irrigation^2a^ - Working time^2a^ - Machine use^2a^ Aggregated biophysical and monetary values - total fuel use^2b^ - Sum of N-, P_2_O_5_- and K_2_O input^2b^ - stand. treatment index^2b^ - Factor costs (total)^2b^ Relational and balancing biophysical values: - Energy use efficiency^3,4a^ - Water use efficiency^3,4b^ - N farm gate balance^2b,4c^ - N soil surface balance^2b,4c^ Indirect inputs: - Development in technology and knowledge ^3^ - Farmers’ education^3^	Non-aggregated, biophysical values - Crop yield^2a^ Non-aggregated, monetary values: - Crop sales2a Aggregated, biophysical values: - Grain equivalent units (total)^2b^ - Grain equivalent units (crops)^2b^ - Grain equivalent units (livestock)^2b^ Aggregated, monetary values: - Sales (total)^2b^ Relational and balancing monetary values: - Income (total)^2b^	Highly integrated /index-coded values Impacts on climate - GHG emissions (CO_2_ equivalent)^2^ Impacts on soil - Erosion by water^3^ - Erosion by wind^3^ - Humus balance^3^ - Soil compaction^3^ Impacts on ground and surface water - Water quantity^3^ - Water quality^3^ Impacts on flora and fauna - Habitat suitability for species of agricultural landscapes (e.g. field birds)^3^ Impacts on cultural ES - Landscape aesthetics^3^ - Recreation^3^	Consumer interests (products) Consumption patterns^3^ - Food consumption (e.g. organic vs. conventional) - Expenses for food Preferences^3^ - Willingness to pay - Willingness to accept Local and regional stakeholder interests (regional ES demand)^3^ Specific preferences for ES of local and regional stakeholders^3^ - Willingness to accept Societal demand (policy strategies)^3^ Indicators belonging to the following mitigation strategies: - greenhouse gas-emissions^3^ - N input into water bodies^3^ - Endangerment of flora and fauna^3^
e.g. single maps, map time series^3^, ‘hot-/ cold-spots’^3^, ‘hot moments’^3^, landscape structure by landscape metrics^3^ and spatial pattern analyses^3^, time and organisational processes of crop cultivation, crop rotation and individual management measures integrated in specific factors of indicators of aspects 1–53				

^1a^Input for bio-economic farm model, ^1b^case study regions (Table 2)

^2a^Quantified by the bio-economic farm model, ^2b^calculation of selected indicators (Table 3)

^3^Not quantified in the case study application

^4^Anthropogenic input and environmental impact (^4a^on climate, ^4b^on water quantity, ^4c^on water quality)

#### ES potential of agro-ecosystems

The ES potential can be characterised by soil and climate conditions. The soil quality rating (Müller et al. 2007) delivers an index which reflects the agronomic yield potential. The rating allows comparisons of the yield potential on a regional level. Precipitation and temperature are important climate factors for the ES potential. Precipitation is also recognised by the soil quality rating as part of the hazard indicator drought.

#### Anthropogenic inputs

The current level of agricultural yields is generally well above the natural production potential of the fields. This level is achieved through the deployment of labour, machinery, water, a number of organic and chemical inputs and energy (direct inputs). This direct input information can be used as separate indicators quantified by biophysical, non-aggregated (e.g. application of specific pesticide product); biophysical, aggregated (e.g. standardised treatment index); monetary, non-aggregated (e.g. costs of specific pesticide product) or monetary aggregated (e.g. total factor costs) values. Furthermore, indicators that relate inputs to realised ES flow (such as energy and water use efficiency) or which reflect balances (e.g. gate balance) can be used. The existing high agricultural productivity levels are also achieved by an emerging level of technology, knowledge and education of farmers (indirect inputs). Further examples for these categories can be found in Table 1.

#### Actual ES flow from agro-ecosystems

The flow of products from agricultural production systems consists of field crop products and livestock products. In a first step, ES flows by crop yields can be indicated as biophysical, non-aggregated (e.g. crop yields) or as monetary, non-aggregated values (e.g. crop sales). In a further step, biophysical, aggregated values can be calculated as a unique indicator value for different kinds of products, the total grain equivalent unit yields of all products that finally leave the farm, related to crop production or related to livestock. Also, total sales as monetary, aggregated values can be calculated. Finally, relational indicators can be used, like total income as monetary values. Further examples for these categories can be found in Table 1.

#### Environmental externalities of provisioning ES

The positive or negative environmental externalities of agricultural production are multifaceted and range from impacts on air, soil and waters to impacts on different flora and fauna in fields, their biodiversity and in the surrounding environment. Also, cultural ES belong to environmental externalities due to the fact that agricultural activities eminently shape cultural landscapes. Therefore, a number of different indicators are needed to capture the impact in the different areas of the ecosystem components: impacts on climate (e.g. GHG emissions expressed as a CO_2_ equivalents, to cover several climate gases), impacts on ground and surface water (e.g. water quantity, water quality), impacts on soil (e.g. soil erosion by water and wind, soil humus balance and soil compaction), impacts on flora and fauna (e.g. habitat suitability for species of agricultural landscapes, like field birds, cf. Glemnitz et al. 2015) and generating aesthetic and recreational values of cultural landscapes. Both, positive and negative externalities can have feedback loops to provisioning ES, since agricultural production also critically depends on ES inputs (e.g. soil formation, water and nutrient cycling, pollination).

#### ES demands and preferences

By definition, ES are used and demanded by the society (Boyd and Banzhaf 2007). Within the society, ES demands and preferences can reflect, first, consumer interests in terms of agricultural products; second, regional and local stakeholder interests which generate a demand in terms of a regional relevant ES supply; and, third, a societal demand which is often taken up by policy strategies and legally binding regulations. That means, demanded ES do not only refer to agricultural products, but include also aspects like the less tangible avoidance of negative externalities such as greenhouse gas emissions, N emissions, biodiversity loss and also cultural ES. Indicators for consumer interests and for regional and local stakeholder interests are mainly a part of socio-economic methods, like analyses of consumption patterns (share of organic and regional food) or preference analyses (WTP-, WTA-analyses).

An example for societal demand is the GHG mitigation strategy that covers emissions from crop and livestock production and which was formalized by International and European climate agreements (United Nations 1998: Kyoto-Protocol)^17^ and by the national policy strategies for instance in Germany (BMU 2016: National Climate Action Plan 2050^18^; BMU 2008: German Adaptation strategy on climate change^19^, and BMU 2014: action programme 2020^20^). Another example reflects the societal aim to reduce N emissions which is regulated by the European Water Directive 2000 (EC 2000)^21^ and national legal regulations in Germany. Furthermore, the complex demand to maintain biodiversity is manifested in international agreements, like the Convention of Biological Diversity (United Nations 1992)^22^, the Fauna Flora Habitat Directive (ECC 1992)^23^ and the National Strategy for Biodiversity in Germany (BMU 2007)^24^. The indicators for societal demands are mainly fixed by legislation and legal regulations, for example, to indicate achievements in water quantity and quality or the status of protected habitats and species indicators that are monitored and reported on the basis of the European Water Framework Directive, respective of the Fauna Flora Habitat Directive.

#### Spatial modelling and mapping of provisioning ES

Spatial modelling and mapping approaches can be applied to analyse and visualise indicators of the before-mentioned aspects. Primarily, such approaches can assess the spatial and temporal variability of these indicators. This can be completed by an analysis of ‘hot and cold spots’ and ‘hot moments’ in order to gain knowledge about the concentration of the different indicators discussed above. Secondarily, specific indicators to explore causal relations and functional linkages can be used. Examples of such specific spatial indicators are landscape metric indicators to assess fragmentation, diversity, habitat connectivity, habitat richness and landscape heterogeneity and geostatistical indicators to describe spatial patterns of all elements of agricultural landscapes (e.g. landscape elements, agricultural used area). Thirdly, temporal indicators to assess the time and organisational processes of crop cultivation, crop rotation and individual management measures of the anthropogenic input can be integrated into equations for evaluating environmental externalities of agricultural activities.

## Methods: case study application of the enhanced indicator set

### Overall methodological approach

To characterise the contribution of specific agricultural land use systems to ES supply, detailed information on the characteristics of the natural environment as well as the anthropogenic inputs was used. In order to test the enhanced indicator set, existing data of a bio-economic modelling approach were used. The bio-economic modelling approach has been applied to three different case study regions in Northern Germany (Spellmann et al. 2017). The model has a large integrated data base regarding land use, i.e. crop-specific agricultural production processes that were used to calculate most of the discussed indicators. It was applied to three case study regions that show a large heterogeneity of agricultural production systems caused by different natural, economic, socio-political and historical conditions (Table 2). The main agricultural land use systems of all three regions were analysed by regarding their current land use with respect to a number of biophysical and monetary indicators at a regional level, with consideration to the heterogeneity of natural site conditions.

**Table 2:** Characterisation of the three northern German study regions Diepholz, Uelzen and Oder-Spree (sources: Statistische Ämter des Bundes und der Länder 2012^1^: data of the year 2010; IACS Lower Saxony 2010/2014^2^; IACS Brandenburg 2010/2014^3^; BGR 2013^4^; DWD 2010^5^)

Characterisation of the regions	Diepholz	Uelzen	Oder-Spree
Federal state	Lower Saxony	Lower Saxony	Brandenburg
Total agricultural used area (ha)^1^	128,701	73,156	78,598
Farms (n)^1^	1969	751	325
Average farm size (ha)^1^	65	97	242
Average regional livestock density (LSU/ha)^1^	1.13	0.28	0.44
Soil Quality (Index of M-SQR, area-weighted) (-)^4^	63	60	51
Average annual temperature 1981-2010, area weighted (°C)^5^	9.5	9.0	9.4
Total annual precipitation 1981-2010, area weighted (mm/a)^5^	728	712	585
Production focus^2,3^	Livestock and biogas	Irrigated potatoes and sugar beets	Cereals and rapeseeds
Share of set-aside of total arable area 2014 (%)^2,3^	1.30	1.30	3.7

### Case study description

The three selected case study regions, in which the proposed indicator set was tested, differ considerably in natural conditions and agricultural structure, which allowed to test the same indicators under different conditions (Table 2). Two of the case study regions (Diepholz and Uelzen) are located in the German federal state of Lower Saxony and one (Oder-Spree) in the state of Brandenburg. Together, the case study regions constitute a transect from western to eastern Northern Germany. The first region, Diepholz, is characterised by high levels of livestock and biogas facilities, relatively good soils and sufficient precipitation. The second region, Uelzen, has poorer soils and less precipitation and is specialised in irrigated potatoes and sugar beet production. The third region, Oder-Spree, has poorer soils as Uelzen and, despite drought problems, almost no irrigated production and a lower level of livestock and biogas facilities; the production is focused on cereals and rapeseed. The case study regions are quite different in structure, agro-environmental conditions and input-output ratios.

### Application of a bio-economic farm model

The bio-economic farm model MODAM (Zander and Kächele 1999; Uthes et al. 2010; Gutzler et al. 2015) was applied to assess various aspects of provisioning ES. To simulate agricultural decision-making under different market and policy conditions, this programming approach was used, because it reflects economic rationality in the decision-making of farmers (Zander and Kächele 1999). This approach uses detailed descriptions of production techniques including all agricultural inputs and the related labour and machinery data. On this basis, the model simulates agricultural income optimisation through mathematical programming. The following steps were conducted:

1. Based on statistical data and interviews with regional farmers and experts, a detailed picture of all crop and production process-specific inputs used by farmers was obtained specifically for each of the three case study regions. In detail, the obtained inputs were:

- Seed amounts which were derived from experts in the regions
- Fertiliser inputs which were calculated according to the nutrient requirements of crops according to the German Fertiliser Ordinance as in force 2010 (DüV 2007), including the maximum allowed surplus of N fertilisation of 60 kg N/ha
- Pest and disease management which was derived from a survey amongst farmers from the research regions (Andert et al. 2015)
- Capacities of biogas plants which were based on the online data of the German Solar Energy Society, DGS, 2012^25^.

2. The production process–related fuel demand, labour and costs for the chosen typical machinery were derived from German agricultural machinery and production and processing data, provided by KTBL (2012, 2013, 2017) and fertilisation by LfL (2013). Product prices were derived from a 3-year average (2008–2010), and subsidies of the CAP regulations from 2010 were applied in the respective regions. Biogas prices and related production restrictions were taken into account according to the charging system in force at the time of the first day of active service of the respective biogas plants.
3. The following endogenous parameters within the farm model were calculated: the roughage for livestock and concentrates from external suppliers, the use of manure, substrate for biogas production, the use of fermentation residues and irrigation water demand. The gross margin of each of the production processes was calculated in an aggregated form for typical regional farms. All calculations were based on the abovementioned data. The calculations of the total gross margins took internal restrictions into account like fodder and substrate production for livestock and biogas plants or the use of manure and digestate.
4. Based on that, an economic optimisation tool was run. The optimisation was based on the total gross margin of the individual production processes. Region-specific land use patterns were derived from this optimisation, i.e. the modelled shares of cultivated crops.
5. The resulting land use patterns of the economic optimisation were used for an aggregation of individual farm data. This was based on the weight of each modelled farm for the region. It resulted in a data basis for the calculation of the selected economic and ecological indicators of the aspects of provision ES.

### Calculation of selected indicators of provisioning agro-ecosystem services

To quantify the different aspects of provisioning ES for the case study regions, a number of indicators from the indicator set (‘Development of the enhanced indicator set for provisioning ES of agroecosystems’, part three) were selected. The main emphasis was placed on the following aspects: anthropogenic inputs, actual ES flow and environmental externalities of provisioning ES (Table 1, quantified indicators in bold). All indicators from the focused aspects are farm model in or outputs. Their quantification is described in the following. Detailed information (rationale, quantification method, data source in) of the used indicators which are aggregated, relational or highly integrated/index-coded indicators is summarised in Table 3.

**Table 3:** Rationale and quantification method of the enhanced indicator set for provisioning ES

Nr	Indicator	Rationale	Quantification method	Sources
1	M-SQR (Muencheberg soil quality rating index)	M-SQR is used here to indicate the agronomic yield potential as given by the arable soils and under local weather conditions (Müller et al. 2007).	The indicator is calculated as the area-weighted average for our study regions of the M-SQR value as provided by the BGR (2013) map that classifies soils of Germany in 6 yield-potential classes from extremely low to very high.	Müller et al. (2007), BGR (2013)
2	Sum of N, P_2_O_5_ and K_2_O input	The nutrient input is used as an indication of the land use intensity of agricultural land use.	The input of N, P_2_O_5_ and K_2_O is calculated as the sum of inputs from mineral fertilisers and bought fodder as calculated by the bio-economic farm model.	MODAM internal calculation
3	STI (standardised treatment index)	The STI is a simple indication of the pesticide use intensity in relation to the recommended dosage. It does not describe the specific impacts of the applications on individual flora and fauna. It delivers a “1” for one treatment at the recommended dosage of one active substance on 100% of the area. A reduced dosage or partial treatment reduces the value (Rossberg et al. 2002).	The STI was calculated for the crop production activities in MODAM which includes pesticide treatments as derived from a survey in the study regions by Andert et al. (2015) and following their methodological approach.	Rossberg et al. (2002), Andert et al. (2015).
4	Total fuel use	The total fuel use within crop production indicates the intensity of mechanical works as part of the anthropogenic input into the agro-ecosystem.	The total energy use of crop production was calculated on the basis of the production processes chosen by the farm economic modelling approach and equals the total diesel consumption in the field and transportation works.	MODAM internal calculation
5	Total factor costs	Total factor costs aggregate the anthropogenic input into the agro-ecosystem in monetary terms and indicates the total invested value into the agro-ecosystem.	The total factor costs were calculated on the basis of the production processes chosen by the farm economic modelling approach as the sum of the costs for operating materials (plant protection agents, fertilisers, etc.), machinery costs, service costs, labour costs.	KTBL (2012)
6	N farm gate balance	N farm gate balance represents the total N losses within a farm (Brouwer 1998). It is a simple indicator of negative external ecosystem impacts of agricultural land use at farm level.	N farm gate balance was calculated as difference between N input into the farm1 and N-output from the farm2 on the basis of the production processes chosen by the economic farm model.	MODAM internal calculation
7	N soil surface balance	N soil surface balance includes the N input which is brought into the field and the N output through yielded products for all areas of the farm (Brouwer 1998).	N soil surface balance was calculated as difference between N input into the farm fields3 and N-export from farm fields4 on the basis of the production processes chosen by the economic farm model.	MODAM internal calculation
8	GEU (Grain equivalent units)	The total grain equivalent units (GEU_total_) indicates the total actual ES flow of the agricultural production system.	The total grain equivalent units were calculated as a sum of the regional derived grain units as the biophysical sold output from crop (indicated by GEU_crops_) and livestock production (indicated by GEU_livestock_) transferred in ‘grain equivalent units’ (BLE/BMELV 2010).	BLE/BMELV 2010
9	CO_2_eq. emission (CO_2_ equivalent emission)	The CO_2_eq emissions from managed agricultural soils and cultures indicate the climate impact of agriculture.	CO_2_eq emission was calculated according to Rösemann et al. (2015); the calculation included the impact of mineral fertilisers, animal manures, digestates from energy plants, grazing, crop residues, CO_2_ from liming, but not the GHG impact of the production of the implementation and was based on a farm model that included production processes.	Rösemann et al. (2015)

^1^N input into the farm (N in mineral fertilisers, N from non-agricultural atmospheric deposition, N fixation by legumes, N in seeds, N in imported fodder)

^2^N export from the farm (N in crop products, N in animal products)

^3^N input into the farm fields (N in mineral fertilisers, N in organic fertilisers, N fixation by legumes, N in seeds, N from agricultural and from non-agricultural atmospheric deposition)

^4^N output from farm fields (N in crop products, N in internal fodder for biogas plants, N in internal fodder for animals)

The ES potential was indicated by the *soil quality rating index (M-SQR)* (Table 3) and thus was used to differentiate the case study regions and relate production processes to these differentiated site conditions.

The anthropogenic input was primarily indicated by non-aggregated biophysical and monetary values (seeds, fertiliser, pesticides, fuel use, irrigation water, working time, machine use) as an integral component of the bio-economic farm model MODAM for the regional relevant production processes (see previous section). And secondly, on this basis, aggregated (*sum of N-, P*_*2*_*O*_*5*_*- and K*_*2*_*O input, standardised treatment index, total fuel use of crop production, total factor costs*) and balancing indicators (*N farm gate balance, N soil surface balance*) were derived (Table 3).

The actual ES flow was primarily indicated by non-aggregated biophysical (crop yield) and monetary values (crop sales) as an integral component of the bio-economic farm model MODAM for the regional relevant production processes (see previous section). Aggregated biophysical (*grain equivalent units of crop production, grain equivalent units of livestock production, total grain equivalent units*) and monetary values were calculated on this basis (Table 3).

The environmental externalities were indicated by greenhouse gas (GHG) emissions (calculated as *CO*_*2*_ *equivalent*). Some of the calculated indicators of anthropogenic input were also used to indicate environmental externalities, like *N farm gate balance*, *N soil surface balance***,** *total fuel use of crop production*, and *standardised treatment index*.

## Results: case study application of the enhanced indicator set

The production systems and intensities in the three different case study regions differ considerably as a result of the differences in natural conditions, farm structures and market access. This is reflected by the income structure, input and output levels and environmental indicators. The farm model results shown in the online resource (Supplement 1) are at a regional level and given in hectare averages. These data are the result of the total regional production generated from a number of typical farms that were weighted by their occurrence in each region. The results show the biophysical quantities for anthropogenic inputs and ES flow and their monetary values.

### Site condition-related ES potential and actual ES flow

**Fig. 5:**
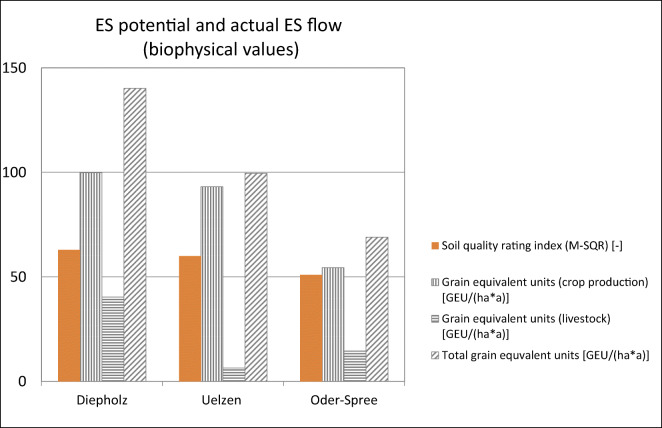
ES potential in terms of soil quality and actual ES flow in terms of grain equivalent units from crop production, livestock and in total (crop production + livestock)

The ES potential, in terms of soil quality (Fig. 5), differs between Diepholz, Uelzen and Oder-Spree, ranging from a higher ES potential in Diepholz and Uelzen to a lower ES potential in Oder-Spree. The actual ES flow in terms of grain equivalent units from crop production reflects this difference, but is also a result of the anthropogenic inputs (see below). Farmers in Diepholz and Uelzen produce similar large amounts of crops of around 100 GEU ha^−1^ a^−1^ (Fig. 5). The large amount of livestock products expressed in grain equivalent units in Diepholz explains the largest total grain equivalent units of around 140 GEU ha^−1^ a^−1^ (Fig. 5) despite a similar ES potential in terms of soil quality. The ES potential in Oder-Spree was lower and crop output was almost half compared to the other two regions. Together with the livestock production, around 70 GEU ha^−1^ a^−1^ was produced in the Oder-Spree region (Fig. 5).

### Anthropogenic inputs into the production system and resulting ES flow (biophysical values)

**Fig. 6:**
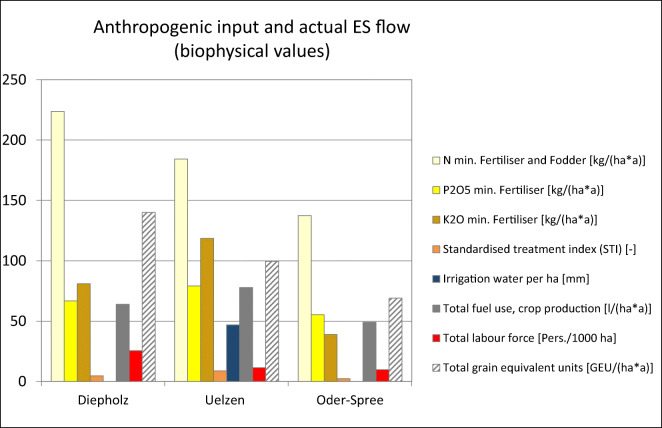
Average anthropogenic inputs in terms of fertilisers, pesticides, water, fuel use and labour as well as actual ES flow in terms of grain equivalent units

The anthropogenic inputs in the three case study regions show different patterns due to the different biophysical and socio-economic conditions. In Diepholz, the region with the highest overall ES flow in terms of crop and animal products, the intensity of production is reflected by the highest inputs of nitrogen of >200 kg N ha^−1^ a^−1^ including fertilisers and fodder, and the highest labour input (Fig. 6). In Uelzen, the inputs for potassium of around 120 kg K_2_O ha^−1^ a^−1^, phosphorus of around 80 kg P_2_O_5_ ha^−1^ a^−1^ and pesticide and fuel use are particularly high due to the intensive cultivation of crops such as sugar beet and potato. Farmers in Uelzen have been able to increase the growth potential of their crops through application of irrigation water. In Oder-Spree, with the lowest production output and ES potential, the overall level of fertiliser inputs was lower with <150 kg N ha^−1^ a^−1^ including fertilisers and fodder, lower potassium and phosphorus inputs and lower fuel and pesticide use compared to the other two regions.

### Anthropogenic inputs into the production system and resulting ES flow (monetary values)

**Fig. 7:**
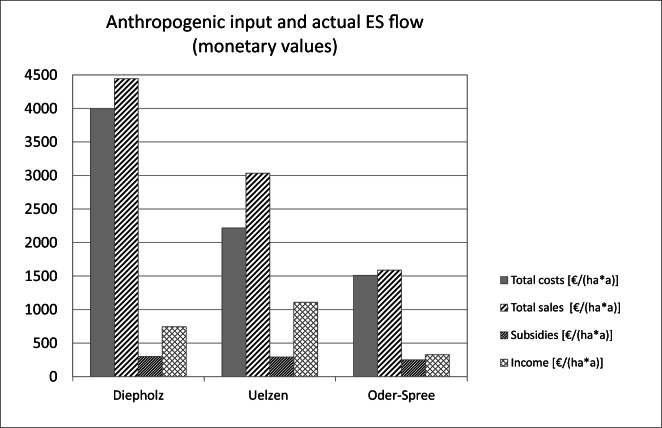
Average income per ha, defined by income = subsidies + (sales − costs), from agricultural production for three regions in Northern Germany (model output)

Similar to the anthropogenic inputs, the total costs decrease from Diepholz to Uelzen and Oder-Spree with 4000 € ha^−1^ a^−1^, with 2200 € ha^−1^ a^−1^, and 1500 € ha^−1^ a^−1^, respectively (Fig. 7). In Diepholz, the livestock sector adds to the total costs per hectare, while costs in Uelzen are dominated by the irrigation-based arable sector with potatoes as the main crop. Costs in Oder-Spree reflect the lowest input level in arable production mainly with cereals and rapeseed.

Subsidies per hectare are similar in all regions with approx. 300 € ha^−1^ a^−1^ (Fig. 7). While sales follow again a decreasing trend from Diepholz in the west to Oder-Spree in the east, farm income does not follow this trend. The high level of inputs with high variable and fixed costs, largely related to milk production in Diepholz, results in an average income of 750 € ha^−1^ a^−1^ (Fig. 7). Despite the high level of inputs, the low price of milk in this scenario leads to lower incomes in comparison to the cash crop-oriented farming in Uelzen. The average income per hectare in Uelzen is the highest with 1100 € ha^−1^ a^−1^, with a production focus on highly profitable arable crops (Fig. 7). The income in Uelzen is almost three times higher than that in Oder-Spree with lower input levels and an income of 300 € ha^−1^ a^−1^ which is only slightly higher than the subsidies (Fig. 7).

### Environmental impacts of provisioning ES: N balance

**Fig. 8:**
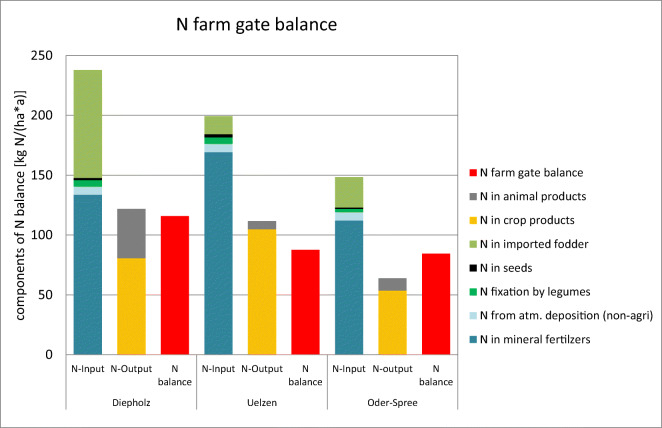
N farm gate balance based on input and output of nitrogen

The N farm gate balance for the three regions reflects very well the intensity levels of the different production systems. A high amount of nitrogen was applied as mineral fertiliser—mainly in the production of maize for fodder and for bioenergy. The N input from this source was comparably high in Uelzen (around 170 kg N ha^−1^ a^−1^) and lower in Diepholz (134 kg N ha^−1^ a^−1^) and in Oder-Spree (112 kg N ha^−1^ a^−1^) (Fig. 8). Additionally, nitrogen from fodder imports is a main N input, especially in Diepholz, with its high livestock density; nitrogen from fodder imports contributes to the highest N input (90 kg N ha^−1^ a^−1^) compared to the other regions (Oder-Spree 25 N ha^−1^ a^−1^, Uelzen 15 kg N ha^−1^ a^−1^) (Fig. 8). N outputs are mainly N exports in crop products, from 105 kg N ha^−1^ a^−1^ in Uelzen to 81 kg N ha^−1^ a^−1^ in Diepholz and 53 kg N ha^−1^ a^−1^ in Oder-Spree and to a minor extent in animal products from 41 kg N ha^−1^ a^−1^ in Diepholz, to 10 kg N ha^−1^ a^−1^ in Oder-Spree and to 7 kg N ha^−1^ a^−1^ in Uelzen (Fig. 8). This leads to an N output between 122 kg N ha^−1^ a^−1^ in Diepholz, 112 kg N ha^−1^ a^−1^ in Uelzen and 64 kg N ha^−1^ a^−1^ in Oder-Spree. The resulting farm gate balance (Fig. 8) thus showed the highest surplus value in Diepholz (116 kg N ha^−1^ a^−1^) compared to values of the other two regions Uelzen (88 kg N ha^−1^ a^−1^) and Oder-Spree (84 kg N ha^−1^ a^−1^) (Fig. 8).

**Fig. 9:**
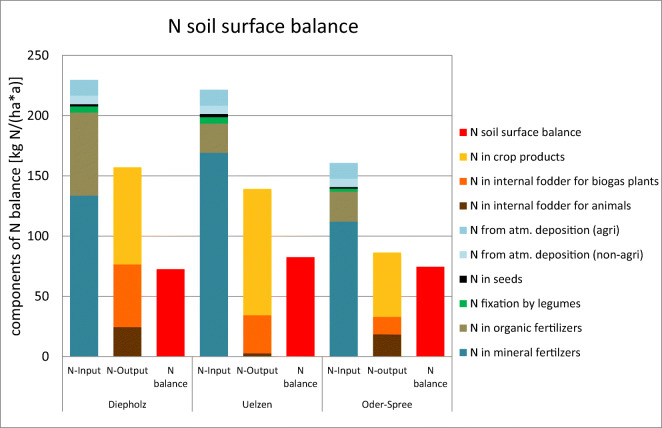
N soil surface balance based on input and output of nitrogen

The N soil surface balance for the three regions shows a picture that is slightly different to the N farm gate balance. The main N input derives from two input sources: from mineral fertilisers which range from the comparable highest value in Uelzen (around 170 kg N ha^−1^ a^−1^) to lower values in Diepholz (134 kg N ha^−1^ a^−1^) and Oder-Spree (112 kg N ha^−1^ a^−1^) (see above) and from organic fertilisers which range the highest in Diepholz (69 kg N ha^−1^ a^−1^) and have lower values in Uelzen and Oder-Spree (around 25 kg N ha^−1^ a^−1^) (Fig. 9). Further N input sources are atmospheric deposition, N fixation by legumes and N in seeds, but all of them are of little importance (Fig. 9). All sources together lead to high and nearly similar N input values in Diepholz (230 kg N ha^−1^ a^−1^) and Uelzen (222 kg N ha^−1^ a^−1^) and to lower values in Oder-Spree (161 kg N ha^−1^ a^−1^) (Fig. 9). The N output is based on three sources in different shares; the N export through crop products differs from 105 kg N ha^−1^ a^−1^ in Uelzen to 81 kg N ha^−1^ a^−1^ in Diepholz and 53 kg N ha^−1^ a^−1^ in Oder-Spree (see above); the N export through internal fodder for biogas plants differs from 52 kg N ha^−1^ a^−1^ in Diepholz to 32 kg N ha^−1^ a^−1^ in Uelzen and 14 kg N ha^−1^ a^−1^ in Oder-Spree; and the internal fodder for animals differs from higher values in Diepholz (24 kg N ha^−1^ a^−1^) and in Oder-Spree (18 kg N ha^−1^ a^−1^) to low values in Uelzen (3 kg N ha^−1^ a^−1^) (Fig. 9). The resulting N soil surface balances show the highest surplus in Uelzen (82 kg N ha^−1^ a^−1^) and lower values in Oder-Spree (74 kg N ha^−1^ a^−1^) and Diepholz (73 kg N ha^−1^ a^−1^) (Fig. 9).

### Environmental impacts of provisioning ES: GHG emissions, pesticide treatment and fuel use

**Fig. 10:**
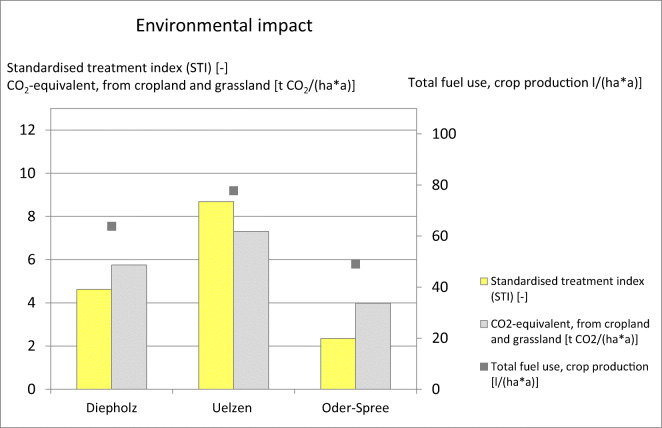
Ecological indicators (greenhouse gas emissions, standardised treatment index for pesticide use and fuel use in crop production)

An overview of ecological indicators, like the greenhouse gas (GHG) emissions from cropland and grassland as a CO_2_ equivalent, an indicator for pest management——the standardised treatment index—in combination with the total fuel use is given in Fig. 10. GHG emissions were only calculated cropland and grassland emissions, not for emissions from livestock. The emitted CO_2_ equivalents of crop production range from about 7 t CO_2_ equivalents ha^−1^ a^−1^ in Uelzen to 6 t CO_2_ equivalents ha^−1^ a^−1^ in Diepholz and 4 t CO_2_ equivalents ha^−1^ a^−1^ in Oder-Spree (Fig. 10). The standardised treatment index was highest in Uelzen (8.7) due to a high pesticide risk because of the large share of sugar beet and potatoes which are treated with a high number of herbicides, insecticides and, especially for potatoes, fungicide treatments. This value was lower in Diepholz (4.6) and Oder-Spree (2.35) due to a lower necessity of plant protection in both regions, especially in Oder-Spree with its large share of rye and other winter crops (Fig. 10). Also, the total fuel use of crop production showed the highest values in Uelzen (78 l ha^−1^ a^−1^) and lower values in Diepholz (64 l ha^−1^ a^−1^) and Oder-Spree (49 l ha^−1^ a^−1^) (Fig. 10).

## Discussion

The development and application of the enhanced indicator set of provisioning ES in agro-ecosystems delivered very useful insights, although the case study application of the indicator set was limited to the chosen modelling approach and the three selected case study regions. Advantages and disadvantages of the enhancement of the indicator set and the application are discussed in the following sections.

### Development of the enhanced indicator set for agro-ecosystem services

The proposed six aspects of agricultural provisioning ES cover ES potential of agro-ecosystems, anthropogenic inputs, actual ES flow from agro-ecosystems, environmental externalities of provisioning ES, ES demand and preferences and spatial modelling and mapping of provisioning ES. Five main characteristics distinguish our approach from others.

First, the enhanced indicator set takes the peculiarities of agro-ecosystems explicitly into account. This has consequences for the data requirements, the initial spatial level of assessment and the upscaling procedure, the types of indicators and the options for scenario applications. It differs from other indicator frameworks that analyse ES for a broad range of ecosystems and ES (cf. Maes et al. 2016; van Zanten et al. 2014). The specific agricultural point of view was, until now, not well-established within the ES concept (Tancoigne et al. 2015).

Second, it is based on ‘data of high-quality requirements’ (Maes et al. 2016, see ‘Development of the enhanced indicator set for provisioning ES of agroecosystems’, part two) that utilise climate and soil data to estimate the ES potential, available crop share and livestock data and additionally, it is based on regional knowledge about production practices. Based on this data, indicators of anthropogenic inputs, ES flow and environmental impact can be calculated, for instance, by applying a farm model like the one used for the three case study regions. The aspect of ES demand and preferences can also be integrated in a farm model, through revealed preferences as given by market prices and through integrating agro-environmental programmes by formulating corresponding restrictions for model calculations.

Third, although the assessment starts at a farm-scale level, it can also be up-scaled to landscape and regional levels. This can be realised through suitable upscaling procedures, e.g. by considering a landscape or region as a larger ‘regional farm’ with a weighted aggregation of the indicators. In this respect, our approach is much different from other indicator frameworks which map ES directly at a much coarser scale such as the landscape (e.g. Ungaro et al. 2014), regional (e.g. Koschke et al. 2013) or national (e.g. Albert et al. 2016) scale. The starting point at a farm scale allows explicitly considering the effects of any agricultural activities on the proposed indicators. Tilman et al. (2002: 676) emphasise the role of farmers as *de facto managers of [...] productive lands [...]*. Thus, the further application of the approach allows, for example, to answer research questions which consider different agricultural management strategies.

Fourth, the indicator set includes five types of indicators, with an increasing level of integration: (i) basic data (site condition, land use data) and (ii) non-aggregated indicators to assess production processes of regional farms, (iii) aggregated indicators to assess aggregated characteristics of regional farms and to facilitate regional comparisons, (iv) relational and balancing indicators to assess resource efficiency and nutrient balances as a key principles for sustainable agriculture (e.g. FAO 2014) and (v) highly integrated, index-coded indicators to assess the environmental externalities of management practices not only on a field scale but also at the landscape and regional level. The quantification can be expressed in biophysical and monetary values and allows to set the results in ecological and socio-economic contexts and to determine direct and indirect valuation (cf. de Groot et al. 2012).

Fifth, the enhanced indicator set offers the option to assess scenario calculations with the proposed indicators, which can be used to compare different policy settings and explore indicator trends for future developments. Furthermore, the proposed framework is an open framework which allows to accommodate further indicators to complement the introduced aspects of provisioning ES.

### Case study application of the enhanced indicator set for agro-ecosystem services

The application of the enhanced indicator set in the three case studies has shown that the consideration of a broad range of different aspects including biophysical and monetary indicators can deliver a comprehensive picture of provisioning ES. Explicit results were shown for ES potential (i.e. soil quality), anthropogenic input (e.g. fertiliser input), ES flow (i.e. crop and livestock products) and environmental externalities (e.g. GHG emissions). Such a detailed indication of agro-ecosystems reduces the risk of misinterpreting land use effects (Albert et al. 2016).

The analysis of the ES potential and the actual ES flow has revealed for the case study regions that the trend of the ES potential, in terms of soil quality rating, and of the actual ES flow, in terms of grain equivalent units from crops, shows the same trend. That means values of both indicators decreased from Diepholz to Uelzen and Oder-Spree (Fig. 5). But it has also revealed that the higher values of grain equivalent units from livestock in Diepholz and Oder-Spree compared to Uelzen did not follow this trend and the grain equivalent units from livestock led to an increase of the total grain equivalent units independent from the ES potential (Fig. 5). This might be related to the fact that the used soil quality rating index (M-SQR) considers the soil quality of arable land (BGR 2013). And a second reason might be that if the livestock farming is based on stable keeping and imported fodder, it becomes more independent from regional fodder production capacity. While the estimation of the potential of soils to supply agricultural provisioning ES by using soil quality ratings (cf. Mueller et al. 2007) provides a quick and useful overview, it can be concluded that such an approach needs to be complemented by an assessment of the actual ES flow and the corresponding anthropogenic inputs. This holds true especially for agricultural systems, which depend to a lower extent on the ES potential, like livestock farming systems with stable systems, farming systems with a high anthropogenic input, e.g. irrigated farming or greenhouse cultivation. Because the used soil quality rating index estimates a productivity potential, the input levels can exploit this potential differently (cf. Mueller et al. 2013). This holds also true for other concepts for soil quality assessments (Bünemann et al. 2018).

The analysis of the anthropogenic inputs and the resulting ES flow in biophysical values has shown that the high total exported grain equivalent units were mainly based on the high N inputs and labour force. In the case studies, the ES flow and inputs were highest in Diepholz and lower in Uelzen and Oder-Spree (Fig. 6). For other inputs, like P_2_O_5_- and K_2_O- fertilisers, standardised treatment index, fuel use for crop production and applied irrigation water, the values were higher in Uelzen than in Diepholz and Oder-Spree (Fig. 6), due to the specific orientation of the regional agricultural systems, i.e. cultivation of potatoes and sugar beets (Table 2). The actual ES flow in terms of monetary values showed a different picture in relation to the monetary assessed input. The sales followed the trend of the total exported grain equivalent units with decreasing values from Diepholz to Uelzen and Oder-Spree (Fig. 7). However, due to high costs of livestock farming, the income in Diepholz was lower than in Uelzen (Fig. 7). In Oder-Spree, the major part of income may originate from subsidies, because total sales were calculated to be only slightly higher than the estimated costs. According to the calculations, farmers in Oder-Spree would not be able to continue production without subsidies in the long run. Within a midterm perspective, farmers can survive such conditions by postponing necessary investments. Thus, our findings show that ES flow and income do not necessarily follow the ES potential; and this has consequences for the indication of actual ES flows. Proxies are often used to describe the actual provision of agricultural ES by land cover–based approaches and by assigning ES supply capacities to specific land cover types (see e.g. Burkhard et al. 2012b). The proxy approach neglects that specific agricultural systems can lead to deviations from the overall trend of a correlation between ES flow and anthropogenic input and such approaches do not take into consideration the differences between biophysical and monetary actual ES flow. The different pictures of ES flow in biophysical and monetary values indicate that the inclusion of monetary values is necessary to get a full picture of provisioning ES. Compared with biophysical ES flow in yield terms, the ES flow, in terms of farm income or sales, is less regarded (Kanter et al. 2018). Some studies already include monetary values, but often not as an integral part of ecologic and economic optimisation (e.g. Koschke et al. 2013). Integrated approaches are possible with bio-economic farm models (Kanter et al. 2018) as used in order to apply the proposed indicator set in this study. Furthermore, such models can help to explore in detail the interactions between ecologic and economic outcomes and thus help to develop strategies for sustainable farming. Furthermore, these models can substantially help to develop environmentally friendly measures with their impacts on economic farm assemblages (costs, sales, income).

The analyses of the N balances have shown a high surplus of N balances in all three case study regions which indicates an intensive agricultural production (van der Zanden et al. 2016). In the three case studies, the N farm gate balance was higher than the N soil surface balance (Figs. 8 and 9), caused by losses at the farmstead and during transport through ammonium and methane emissions from manure and digestate. This leads to differences especially for regions with a higher share of livestock farming, such as Diepholz in our study. This means the surplus of the N farm gate balance followed the trend of the ES flow in terms of exported total grain equivalent units (Fig. 8 compared to Fig. 5), but the soil surface balance did not follow this trend (Fig. 9), because this balance approach cannot depict the N losses at the farmstead and during transport (see above). Furthermore, a comparison of the N farm gate balances between Uelzen and Oder-Spree shows that the N balance in Oder-Spree was only slightly lower, despite a significant lower ES flow (Fig. 9). Overall, the high N surplus that was found in all three case study regions reflects the high intensity of the production systems that are standard in central Europe (Tilman et al. 2002, 2011; Rockström et al. 2017). A positive N balance value usually indicates that N is gained in the system, and a negative value indicates losses and implies that all sources, sinks and losses of N are accounted for in the calculation (Sainju 2017). However, in this study, not all N outputs were included in the calculation of the N balance, and therefore, especially losses via N leaching could not be estimated. A high N balance in this study indicates large losses to the environment and occurs as a negative externality. A positive externality and N balance could also indicate a ‘service’, which can be achieved by adapted management measures (Martinho 2019) to mitigate N losses or to even build up soil fertility (N is added to the soil N pool) (Sainju 2017) due to conservation practices and legume production (Reckling et al. 2016).

The other environmental externalities were calculated only for crop production and considered GHG-emissions, fuel use and pesticide treatment (Fig. 10). The first two indicators are related to the urgent demand for mitigating climate change effects (Burney et al. 2009; Lal et al. 2011). The latter one is related to the demand for efforts towards avoiding side effects of plant protection measures (e.g. on water quality and non-target species) for which integrated management approaches exist (e.g. Barzman et al. 2015). In this sense, they indicate externalities of production processes related to regulating and habitat ES. GHG emissions, fuel use, and pesticide treatment indicators showed higher values for Uelzen than for Diepholz and especially Oder-Spree (total fuel use, CO_2_ equivalent, standardised treatment index), although the ES potential and actual ES flow in terms of grain equivalent units exported from crop production were not as high as in Diepholz, but higher than in Oder-Spree (Fig. 10). This was caused by the specific agricultural structure in Uelzen and depends on the needs of the cultivated crops (Table 2), i.e. pesticide treatment needs and high fuel input for cultivating potatoes and sugar beets. However, the high livestock share in Diepholz contributed as well to GHG and its consideration would change the picture in favour of Uelzen.

The results show that not only the ES potential can lead to different actual ES flows due to a different indication of the output and different production systems. The indication of outputs, i.e. whether to indicate the actual ES flow by grain equivalent units or by income parameters, plays a role for the valuation of the actual ES flow. An example is that for farmers in Diepholz, with a focus on livestock farming, the highest ES flow was calculated in terms of biophysical output but not in terms of income. Instead, for the farmers in Uelzen, the highest ES flow was calculated in terms of income by producing crops with higher gross margins, which need high long-term investments in irrigation technology and marketing instruments. Another example is that the current difference in production intensity between Uelzen and Oder-Spree cannot be solely traced back to ES potential, but has also market-structural reasons based on historical developments. Both regions developed in different markets (Western and Eastern Germany, respectively) until the end of the 1980s. The expensive irrigation practices in Uelzen were propelled by attractive contracts for potato production with market demands for certain product qualities and hence intensified the production of potatoes. Nowadays, irrigation would be possible in Oder-Spree as well, which could lead to a compensation of the low ES potential of Oder-Spree in terms of crop yield. However, after the German reunification in 1990, this strategy was abandoned due to the limited access to capital for East German farmers to reinvest and due to the market power, which established potato-producing farmers were able to generate and which hindered the market entry of newcomers. Both examples show the importance of the specific agricultural production structures that depend not only on natural conditions but also on factors, like market prices for produced goods, established market access, investments made in the past, investment power and historical background.

The results of the case study application revealed that a more detailed consideration of the agricultural production system in a certain region, which makes provisioning ES available, helps to enhance the assessment of the current situation. It could be shown that only regarding an ES potential or proxies for ES supply, like land cover types, can lead to misinterpretations by neglecting the regional specifics of an agricultural system. Furthermore, such approaches do not allow a detailed consideration of different management options, which are necessary, for example, to improve management strategies towards an economically viable and environmentally and biodiversity-friendly agricultural production. The full explanatory power of the enhanced indicator set for provisioning ES can be achieved only by using the indicator set for answering further specific research questions. These questions determine also which aspects and indicators are focused and weighted more heavily.

Uncertainties of the suggested indicator set and its application

### Uncertainties of the suggested indicator set and its application

Uncertainties of the suggested indicator set are linked to:

(i) Data quality issues: The data sources for the advocated indicators in Table 1 can normally be based on agricultural census of cultivated crops (see Fig. 4) with a spatial resolution on county level (equivalent to NUTS-3-level^26^), a temporal resolution of several year periods and a thematic resolution based on main cultivated crops. Data of anthropogenic inputs are usually not directly available with a resolution on county level or finer, because they are normally assessed as census data on state level and are kept in FADN^27^ datasets as data on fertiliser use per farm but not per crop. Furthermore, these data cover only a number of typical farms, of which the representativeness can be questioned on a regional and local level. Data of ES demands and preferences are often part of valuation panels or specific studies with an inherent limited duration of validity and limited spatial transferability. In the case studies, calculations were carried out with a bio-economic farm model, based on farm structural data as assessed from crop specific land use data (IACS data) and input-output relations for all production activities based on KTBL data and local experts. Regional knowledge about production practices of the cultivated crops, yield statistics and expert interviews to reconstruct site specific crop production activities were used. The farm model allowed us to take into account farm internal interrelations of manure and biogas digestate with crop production and mineral fertiliser use. Information about demand and preferences was integrated indirectly into the calculation restrictions of the farm model.
(ii) Methodological standards: Schulp et al. (2014) stated a need for standardisation of the methods for indicator derivation and indicator data sources in the context of environmental impact. The suggested indicators of the proposed indicator set are not yet standardised, but they are ready to be used for impact analyses. However, the more comprehensive and detailed an indicator set becomes, the more effort and data are needed for application. Thus, compromises often need to be made between what we actually want to know and what is feasible in a certain study context and with available resources. Nevertheless, further applications and improvements are of course feasible. Especially further indicators, which assess the site-specific interrelations between land management and biodiversity-related ES like pollination, pest and disease control (e.g. Tamburini et al. 2016) or the control of water quality and soil erosion (e.g. Sattler et al. 2010; Albert et al. 2016), are needed.
(iii) Completeness of the indicator set: The indicator set does not yet contain indicators for functional agricultural biodiversity, (cf. Moonen and Barberi 2008; Bianchi et al. 2013), nor indicators that relate directly to the demand and supply side of provisioning ES, which, for example, can be done by using ES footprints (Burkhard et al. 2012b). Also, indicators for economic and ecologic resilience are still missing as in many other indicator-based evaluation approaches (cf. Kanter et al. 2018), despite their growing importance (Ge et al. 2016; Peterson et al. 2018).

## Conclusions

### Rationale and application of the enhanced indicator set

Our approach confirms the statement of Saunders et al. (2018: p. 389) who stress that ‘the concept of ES is not about humans passively receiving benefits from “wild” nature’. Our suggested indicator set enables to assess the ES supply as a combined outcome of natural ecosystem potentials and anthropogenic inputs. In our case study application, we showed that only a combined assessment of ES potential and anthropogenic inputs could appropriately explain the realised ES supply, whereby the whole farm management system can actually be seen as an anthropogenic input. From this point of view, the indicator set can accompany regional and local improvements in environmental, biodiversity-friendly management measures as a part of an informed management strategy (Pérez-Soba et al. 2012).

Furthermore, the indicator set allows to relate single aspects to each other, for example, ES flow *versus* anthropogenic inputs or environmental impact *versus* anthropogenic inputs. The approach also integrates monetary values which are essential for the assessment of economic outcomes, based on monetary analyses of anthropogenic inputs and ES flow. The approach integrates biophysical, non-monetary values which are essential for the ecological assessment of anthropogenic inputs and of the ES flow and its environmental externalities and for comparisons of ES supply with demand.

The indicator set is suitable for practical applications at case study level, as was shown by the application in the three case studies. The basic data for the indicator set are generally available and enable to measure the proposed six aspects. They serve the different levels of the indicators, which fulfil specific requirements, for example, comparing regions through aggregated indicators and assessing resource efficiency through relational indicators and environmental externalities through index-coded indicators, which are mainly based on agricultural production processes. Additional indicators can expand the proposed indicator set, as they can be assigned to the six aspects.

For a wider application of the indicator set in a European/international context, the dataset can be transferred in terms of data availability and measurability of all aspects of the indicator set. The basic data are a part of agricultural census and generally available, and the calculation of other indicators is based on them. Therefore, all aspects can be quantified in other regions. In general, all indicators of the enhanced indicator set allow an upscaling, so the aggregated and the highly integrated/index-coded indicators can monitor ES supply of landscapes and therefore support landscape-oriented, collaborative decision-making processes.

### Contributions of the enhanced indicator set for understanding and indication of the interrelations between agriculture, ecosystems and landscapes

The understanding of the interrelations between agricultural production, ecosystems and the landscape context can be clarified by distinguishing the six aspects of the indicator set. The indicators of the proposed set are scalable from farms to agricultural landscapes and regions. Landscape orientation is a new approach for agricultural research and for approving new management strategies at a landscape level (Wolters et al. 2014). The research questions of this field concern resource efficiency at a landscape level, landscape-specific definitions of production aims, design of landscape elements, impact assessment and management adaption of production inputs on biodiversity components at a landscape level (ibid). Therefore, new assessment schemes and indicator sets are needed to accompany agricultural research and should be based on a systemic approach and an integration of economic and ecologic aspects (ibid).

### Recommendations for improvements of the enhanced indicator set

Based on the uncertainties of the suggested indicator set (see ‘Discussion’, part three), improvements concern (i) data quality issues, (ii) methodological standards and (iii) completeness of the indicator set.

(i) Data quality issues: The data availability in our approach concerns mainly the agricultural census crop cultivation data, which could for instance be augmented by data from the Integrated Administration and Control System (IACS, cf. Kandziora et al. 2013a) with a high spatio-temporal and thematic resolution. The IACS data are assessed from farms in European Union member states that apply for agricultural European subsidies, refer to a field scale, are assessed on a yearly basis and include all cultivated crops, instead of the availability on a county scale for a several year period and main cultivated crops only of the agricultural census data. The accessibility of data of anthropogenic inputs and of ES demands and preferences cannot be easily improved, due to the fact that the collection of both of these data would be time and resource consuming, due to a high regional and temporal variability of consumer demands and preferences.
(ii) Methodological standards: Indicators could be improved by the integration of interrelations between management and landscape by using spatially explicit data of biotope and landscape structures and combining them with the management information to calculate local and regional effects on biodiversity aspects, water and soil.
(iii) Completeness of the indicator set: The indicator set is open for an implementation of new indicators, such as indicators for functional agricultural biodiversity (cf. Moonen and Barberi 2008; Bianchi et al. 2013), indicators that relate the demand and supply side of provisioning ES (Burkhard et al. 2012b) and indicators of economic and ecologic resilience with a scope to resource scarcity, climate change and other future trends (cf. Peterson et al. 2018). Such applications could help to enhance, for example, the adaptability at farm level, transformability at regional level and food security at a global level (Ge et al. 2016).

Improvements of the data sources and of the indicators, as well as developments and integration of further indicators into the proposed aspects of provisioning ES, could enhance the scope and the applicability of the indicator set.

The enhanced indicator set of provisioning ES in agro-ecosystems can help to better understand and analyse complex agricultural ES co-production schemes and their effects on the environment. The indicator set will certainly need to be adapted for practical applications, especially in regard to the availability of suitable (optimum) data on relevant spatio-temporal scales. If applied correctly, the indicator set can support the development of sustainable site-specific agricultural land management strategies that make use of natural conditions while reducing anthropogenic inputs and negative effects on the environment. In general, the indicator set can contribute to evidence-based decision-making processes on different scales.

## Supplementary information

ESM 1 Indication of provisioning ES in the three study regions Diepholz, Uelzen, Oder-Spree. (DOCX 26 kb)

## Footnotes

1 http://ec.europa.eu/environment/nature/biodiversity/strategy/index_en.htm

2 https://www.whitehouse.gov/sites/whitehouse.gov/files/omb/memoranda/2016/m-16-01.pdf (Memorandum for executive departments and agencies (2015)

3 SDG 15: protect, restore and promote sustainable use of terrestrial ecosystems, sustainably manage forests, combat desertification and halt and reverse land degradation and halt biodiversity loss: https://sustainabledevelopment.un.org/sdg15

4 https://www.millenniumassessment.org/documents/document.356.aspx.pdf

5 http://wedocs.unep.org/bitstream/handle/20.500.11822/20120/TEEB_Synthesis_Report_2010.pdf

6 http://cices.eu/

7 http://www.coe.int/en/web/landscape/home

8 Agro-ecosystems have been defined as highly managed ecosystems that are both providers and consumers of ES, mainly designed to provide food, forage, fibre, bioenergy and pharmaceuticals (Power 2010).

9 http://ec.europa.eu/environment/nature/biodiversity/comm2006/2020.htm

10 http://biodiversity.europa.eu/maes

11 https://ec.europa.eu/info/sites/info/files/food-farming-fisheries/key_policies/documents/cap-post-2020-environ-benefits-simplification_en.pdf

12 System of Environmental-Economic Accounting and Experimental Ecosystem Accounting; framework available from https://seea.un.org/sites/seea.un.org/files/seea_eea_final_en_1.pdf

13 http://ec.europa.eu/environment/integration/research/newsalert/pdf/natural_capital_accounting_taking_stock_IR16_en.pdf

14 http://ec.europa.eu/environment/water/water-framework/index_en.html

15 https://ec.europa.eu/environment/nature/legislation/habitatsdirective/index_en.htm

16 https://ec.europa.eu/info/food-farming-fisheries/key-policies/common-agricultural-policy/financing-cap/controls-and-transparency/managing-payments_en

17 https://unfccc.int/sites/default/files/kpeng.pdf

18 https://www.bmu.de/themen/klima-energie/klimaschutz/nationale-klimapolitik/klimaschutzplan-2050/

19 https://www.bmu.de/download/deutsche-anpassungsstrategie-an-den-klimawandel/

20 https://www.bmu.de/publikation/aktionsprogramm-klimaschutz-2020/

21 http://ec.europa.eu/environment/water/water-framework/index_en.html

22 https://www.cbd.int/doc/legal/cbd-en.pdf

23 https://ec.europa.eu/environment/nature/legislation/habitatsdirective/index_en.htm

24 https://www.bmu.de/themen/natur-biologische-vielfalt-arten/naturschutz-biologische-vielfalt/allgemeines-strategien/nationale-strategie/

25 https://www.dgs.de/aktuell/

26 Nomenclature des Unités Territoriales Statistiques of the European Union

27 Farm Accountancy Data Network
