# Supplementary material for: Towards an enhanced indication of provisioning ecosystem services in agro-ecosystems
Source: Environ Monit Assess. 2021 May 14;193(Suppl 1):269. doi: 10.1007/s10661-020-08816-y (PMC8121745; doi:10.1007/s10661-020-08816-y)
Supplement: Supplementary file 1 — Indication of provisioning ES in the three study regions Diepholz, Uelzen, Oder-Spree. (DOCX 26 kb) [file 10661_2020_8816_MOESM1_ESM.docx]

**Online resource: supplement 1**

**Article title:** Towards an enhanced indication of provisioning ecosystem services in agro-ecosystems

**Journal name:** Environmental Monitoring and Assessment

**Author names:** Claudia Bethwell*, Benjamin Burkhard, Katrin Daedlow, Claudia Sattler, Moritz Reckling, Peter Zander

*Corresponding author: Leibniz Centre for Agricultural Landscape Research ZALF, email: claudia.bethwell@zalf.de

Table online resource: supplement 1 Indication of provisioning ES in the three study regions Diepholz, Uelzen, Oder-Spree.

| **Indicator** | **Diepholz** | **Uelzen** | **Oder-Spree** | **Unit** |
| --- | --- | --- | --- | --- |
| **Livestock** | | | | |
| livestock unit (LU)^1^ | 1,02 | 0,14 | 0,39 | [LU ha^-1^ a^-1^] |
| **Seeds** | | | | |
| Total amount of seeds | 338,52 | 617,43 | 93,66 | [kg ha^-1^ a^-1^] |
| **Nitrogen fertilization and balance** | | | | |
| N in mineral fertilzers | 133,57 | 169,28 | 112,11 | [kg ha^-1^ a^-1^] |
| N total before spreading manure | 0,80 | 0,17 | 1,31 | [kg ha^-1^ a^-1^] |
| N total before spreading digestate | 79,10 | 28,13 | 27,76 | [kg ha^-1^ a^-1^] |
| N total before spreading organic fertilizer | 79,90 | 28,29 | 29,07 | [kg ha^-1^ a^-1^] |
| N loss from spreading manure | 0,08 | 0,02 | 0,13 | [kg ha^-1^ a^-1^] |
| N loss from spreading digestate | 10,82 | 4,03 | 4,10 | [kg ha^-1^ a^-1^] |
| N loss from spreading organic fertilizer | 10,90 | 4,05 | 4,23 | [kg ha^-1^ a^-1^] |
| N loss from storage manure for spreading | 0,34 | 0,07 | 0,56 | [kg ha^-1^ a^-1^] |
| N loss from storage manure for biogas | 13,16 | 2,09 | 3,22 | [kg ha^-1^ a^-1^] |
| N loss from storage organic fertilizer | 13,50 | 2,16 | 3,78 | [kg ha^-1^ a^-1^] |
| N in manure without losses | 1,15 | 0,24 | 1,87 | [kg ha^-1^ a^-1^] |
| N in digestate without losses | 92,26 | 30,22 | 30,98 | [kg ha^-1^ a^-1^] |
| N in organic fertilizer without losses | 93,41 | 30,46 | 32,84 | [kg ha^-1^ a^-1^] |
| N in organic fertilizers with losses | 69,01 | 24,25 | 24,83 | [kg ha^-1^ a^-1^] |
| N in organic fertilizers per fertilized area without losses | 148,53 | 139,14 | 95,49 | [kg ha^-1^ a^-1^] |
| Atmospheric deposition (NOx) | 7,00 | 7,00 | 7,00 | [kg ha^-1^ a^-1^] |
| Atmospheric deposition (NHy) | 13,00 | 13,00 | 13,00 | [kg ha^-1^ a^-1^] |
| N fixation of legumes | 5,24 | 5,26 | 2,60 | [kg ha^-1^ a^-1^] |
| N from seeds | 1,83 | 2,73 | 1,26 | [kg ha^-1^ a^-1^] |
| N in internal fodder for animals | 24,47 | 2,75 | 18,48 | [kg ha^-1^ a^-1^] |
| N in internal fodder for biogas | 51,99 | 31,61 | 14,39 | [kg ha^-1^ a^-1^] |
| N in imported fodder | 90,15 | 14,98 | 25,39 | [kg ha^-1^ a^-1^] |
| N export in crop products | 80,66 | 104,71 | 53,48 | [kg ha^-1^ a^-1^] |
| N export in animal products | 41,22 | 6,94 | 10,45 | [kg ha^-1^ a^-1^] |
| N soil surface balance input | 229,64 | 221,51 | 160,81 | [kg ha^-1^ a^-1^] |
| N soil surface balance output | 157,12 | 139,07 | 86,35 | [kg ha^-1^ a^-1^] |
| N soil surface balance | 72,52 | 82,45 | 74,46 | [kg ha^-1^ a^-1^] |
| N farm gate balance input | 237,79 | 199,25 | 148,37 | [kg ha^-1^ a^-1^] |
| N farm gate balance output | 121,88 | 111,65 | 63,93 | [kg ha^-1^ a^-1^] |
| N farm gate balance | 115,90 | 87,60 | 84,44 | [kg ha^-1^ a^-1^] |
| **Other fertilizer nutrients input** | | | | |
| P2O5 in mineral fertilizers | 66,61 | 79,09 | 55,37 | [kg ha^-1^ a^-1^] |
| P2O5 from organic fertilization | 38,55 | 12,15 | 11,85 | [kg ha^-1^ a^-1^] |
| P2O5 from organic fertilization per fertilized area | 61,32 | 55,52 | 34,67 | [kg ha^-1^ a^-1^] |
| K2O in mineral fertilisers | 80,88 | 118,51 | 38,85 | [kg ha^-1^ a^-1^] |
| K2O from organic fertilization | 88,11 | 31,14 | 38,70 | [kg ha^-1^ a^-1^] |
| K2O from organic fertilization per fertilized area | 140,10 | 142,27 | 112,51 | [kg ha^-1^ a^-1^] |
| MgO in mineral fertilisers | 41,66 | 45,96 | 12,82 | [kg ha^-1^ a^-1^] |
| MgO from organic fertilization | 22,24 | 6,31 | 7,12 | [kg ha^-1^ a^-1^] |
| MgO from organic fertilization per fertilized area | 35,36 | 28,83 | 20,70 | [kg ha^-1^ a^-1^] |
| **Pesticides** | | | | |
| Standardised treatment index (STI)^2^ | 4,62 | 8,69 | 2,35 | [-] |
| **Irrigation water** | | | | |
| irrigation water | 0,00 | 468,13 | 0,00 | [m³ ha^-1^ a^-1^] |
| **Energy** | | | | |
| Total fuel use in crop production | 63,84 | 77,77 | 49,00 | [l ha^-1^ a^-1^] |
| **Labour input** | | | | |
| Total labour force (persons) per 100 ha | 2,56 | 1,15 | 0,98 | [pers. 100 ha^-1^ a^-1^] |
| Total hours labour in crop production per ha | 6,27 | 12,18 | 4,24 | [h ha^-1^ a^-1^] |
| **Other output and impact indicators** | | | | |
| CO_2_-equivalent emissions from grass- and cropland^3^ (CO_2_eq) | 5,75 | 7,31 | 3,97 | [t CO2eq ha^-1^ a^-1^] |
| Grain equivalent units, livestock (GEU_livestock_)^4^ | 40,38 | 6,42 | 14,58 | [GEU ha^-1^ a^-1^] |
| Grain equivalent units, crop production (GEU_crops_)^4^ | 99,76 | 93,06 | 54,43 | [GEU ha^-1^ a^-1^] |
| Total grain equivalent units (GEU_total_)^4^ | 140,14 | 99,48 | 69,00 | [GEU ha^-1^ a^-1^] |
| Biogas production | 4524,14 | 2577,73 | 1450,36 | [kWh ha^-1^ a^-1^] |
| **Costs** | | | | |
| Costs of pesticides | 170,55 | 289,88 | 88,23 | [€ ha^-1^ a^-1^] |
| Total costs of work | 531,75 | 474,25 | 308,65 | [€ ha^-1^ a^-1^] |
| Total costs of production (fix, variable, overhead) | 3998,58 | 2217,18 | 1510,15 | [€ ha^-1^ a^-1^] |
| **Economic output** | | | | |
| Revenues per ha | 4443,88 | 3033,59 | 1590,03 | [€ ha^-1^ a^-1^] |
| Premiums per ha | 299,74 | 292,39 | 249,57 | [€ ha^-1^ a^-1^] |
| Net Value added at factor cost per ha | 745,03 | 1108,80 | 329,45 | [€ ha^-1^ a^-1^] |

^1^**LU** - Livestock Unit: Measurement for the number of animals (KTBL 2017)

^2^**STI** - Standardised Treatment Index to measure the pesticide use intensity. The STI is a simple indication of the pesticide use intensity in relation to the recommended dosage. It does not describe the specific impacts of the applications on individual flora and fauna. It delivers a “1” for one treatment at the recommended dosage of one active substance on 100% of the area. A reduced dosage or partial treatment reduces the value (Rossberg et al. 2002).

^3^**CO_2_eq** - CO_2_-equivalent emissions from managed agricultural soils and cultures: Output in CO_2_ equivalent, includes impact of mineral fertilisers, animal manures, digestates from energy plants, grazing, crop residues, CO_2_ from liming but not the GHG impact of the production of the implements (Rösemann et al. 2015) N in imported fodder per ha (LfL 2013) N export in animal products per ha: (LfL 2013) N export in crop products per ha.

^4^**GEU** - Grain Equivalent Units: all natural sold output, like grains, meat, milk etc. is transferred in ‘grain equivalent units‘ (BLE/ BMELV 2010) as Grain equivalent units from livestock (GEU_livestock_), Grain equivalent units from crop production (GEU_crops_), Grain equivalent units, total (GEU_total_)
